# Podocyte-specific Crb2 knockout mice develop focal segmental glomerulosclerosis

**DOI:** 10.1038/s41598-021-00159-z

**Published:** 2021-10-15

**Authors:** Akiko Tanoue, Kan Katayama, Yugo Ito, Kensuke Joh, Masaaki Toda, Taro Yasuma, Corina N. D’Alessandro-Gabazza, Hiroshi Kawachi, Kunimasa Yan, Masaaki Ito, Esteban C. Gabazza, Karl Tryggvason, Kaoru Dohi

**Affiliations:** 1 Department of Cardiology and Nephrology, Mie University Graduate School of Medicine, 2-174 Edobashi, Tsu, Mie 514-8507 Japan; 2 Department of Medical Biochemistry and Biophysics, Karolinska Institute, Stockholm, Sweden; 3 Department of Pediatrics, Kyorin University School of Medicine, Tokyo, Japan; 4 Department of Pathology, The Jikei University School of Medicine, Tokyo, Japan; 5 Department of Immunology, Mie University Graduate School of Medicine, Mie, Japan; 6 Department of Cell Biology, Kidney Research Center, Niigata University Graduate School of Medical and Dental Sciences, Niigata, Japan; 7 Cardiovascular and Metabolic Disorders Program, Duke-NUS Medical School, Singapore, Singapore

**Keywords:** Genetics, Nephrology

## Abstract

Crb2 is a cell polarity-related type I transmembrane protein expressed in the apical membrane of podocytes. Knockdown of *crb2* causes glomerular permeability defects in zebrafish, and its complete knockout causes embryonic lethality in mice. There are also reports of Crb2 mutations in patients with steroid-resistant nephrotic syndrome, although the precise mechanism is unclear. The present study demonstrated that podocyte-specific Crb2 knockout mice develop massive albuminuria and microhematuria 2-month after birth and focal segmental glomerulosclerosis and tubulointerstitial fibrosis with hemosiderin-laden macrophages at 6-month of age. Transmission and scanning electron microscopic studies demonstrated injury and foot process effacement of podocytes in 6-month aged podocyte-specific Crb2 knockout mice. The number of glomerular Wt1-positive cells and the expressions of Nphs2, Podxl, and Nphs1 were reduced in podocyte-specific Crb2 knockout mice compared to negative control mice. Human podocytes lacking CRB2 had significantly decreased F-actin positive area and were more susceptible to apoptosis than their wild-type counterparts. Overall, this study's results suggest that the specific deprivation of Crb2 in podocytes induces altered actin cytoskeleton reorganization associated with dysfunction and accelerated apoptosis of podocytes that ultimately cause focal segmental glomerulosclerosis.

## Introduction

Focal segmental glomerulosclerosis (FSGS) is a clinicopathological disease characterized by steroid-resistant nephrotic syndrome (SRNS) that eventually leads to end-stage kidney disease in the majority of patients, while some FSGS patients are responsive to steroids^1^. The Columbia classification of FSGS includes five pathologic variants: perihilar, cellular, tip, collapsing, and not otherwise specified FSGS^2^. This classification is useful for predicting prognosis^3^. Patients with the tip variant have a good prognosis, whereas cases with the collapsing variant have a poor prognosis^3^. However, the current classification does not include genetic information. Six forms of FSGS, including primary, adaptive, APOL1-associated, genetic, medication-associated, and viral FSGS, have been proposed^4^. Next-generation sequencing of 24 genes associated with SRNS were useful for detecting mutations^5^. Furthermore, about 30% of SRNS cases were reported to be caused by a single gene among 27 genes^6^. Next-generation sequencing techniques were highly effective for detecting FSGS caused by abnormalities of podocyte genes^7^.

Crumbs were first identified as a neurogenic gene containing epidermal growth factor (EGF)-like repeats in *Drosophila melanogaster*^8^. Crumbs were expressed along the entire apical membrane of Drosophila's epithelial cells^9^. They are important for epithelial cell polarity^10^. Crumbs is a type I transmembrane protein containing a large extracellular domain, a transmembrane domain, and a cytoplasmic domain. The short cytoplasmic domain of Crumbs interacts with moesin, an actin-binding protein^11,12^. The human orthologue of Crumbs is CRB1 and mutations in CRB1 cause retinitis pigmentosa^13^.

In humans, the Crumbs family consists of CRB1, CRB2, and CRB3, and the three members share a FERM (4.1 protein, Ezrin, Radixin, Moesin)-binding domain in the cytoplasmic domain^14^. CRB1 and CRB2, but not CRB3, contain EGF and Laminin A/G domains in the extracellular domain. CRB2 is also an evolutionarily conserved gene, and knockdown of the zebrafish orthologue crb2b with morpholino lead to pericardial edema and glomerular permeability defects^15^. Furthermore, full knockout of Crb2 in mice caused embryonic lethality^16^. There have been reports showing induction of SRNS by CRB2 mutations, although the precise mechanism remains unclear^17,18^. These previous observations pointed to the CRB2 gene as a single causative gene of SRNS, therefore in the present study, we generated podocyte-specific Crb2 knockout mice using the Cre-loxP system.

## Results

### Generation of podocyte-specific Crb2 knockout mice

**Figure 1:**
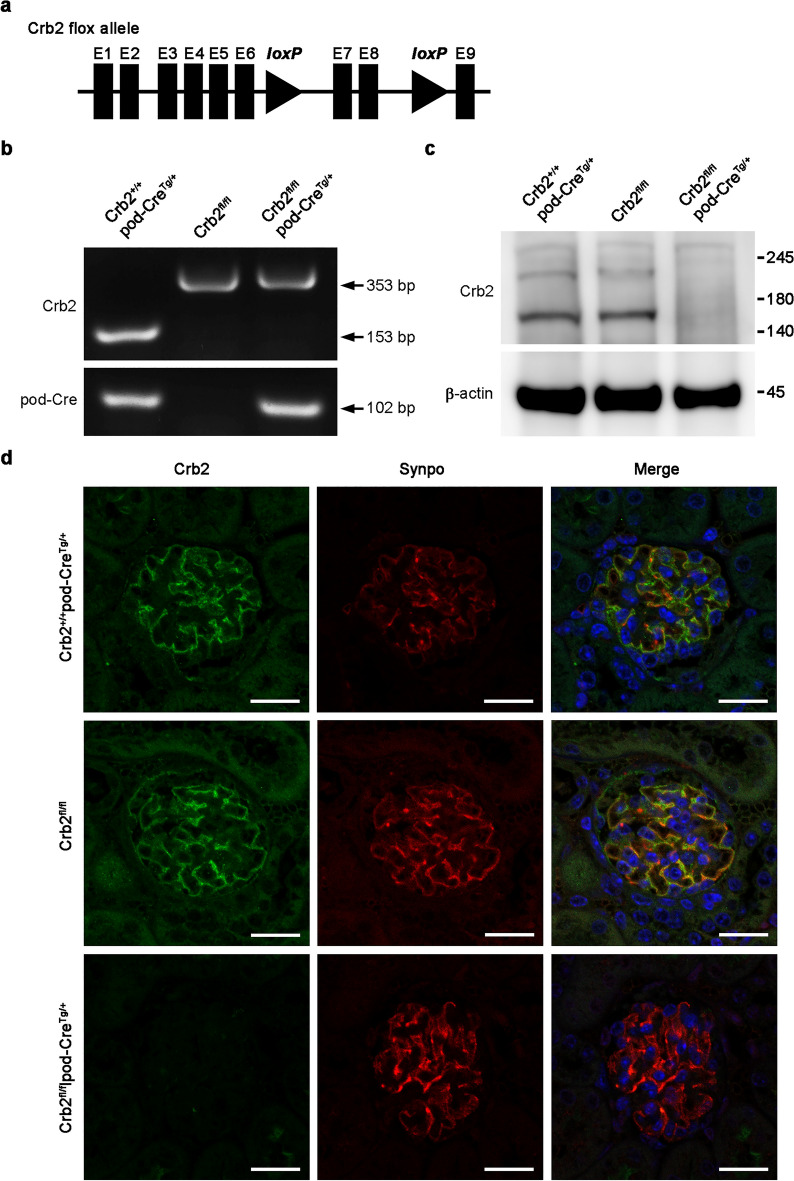
Generation of podocyte-specific Crb2 knockout mice. (**a**) Two loxP sites were inserted into introns 6 and 8 of the Crb2 gene to skip exons 7 and 8 in the Crb2 floxed allele. (**b**) While there was a 353-bp band with Crb2 primers in Crb2^fl/fl^ mice and Crb2^fl/fl^pod-Cre^Tg/+^ mice, there was a 153-bp product in Crb2^+/+^pod-Cre^Tg/+^ mice. There was a 102-bp band with pod-Cre primers in Crb2^+/+^pod-Cre^Tg/+^ mice and Crb2^fl/fl^pod-Cre^Tg/+^ mice. (**c**) There were a strong band between 140–180 kDa and a weak band between 180–245 kDa markers with CRB2 antibody in the glomeruli from Crb2^+/+^pod-Cre^Tg/+^ mice or Crb2^fl/fl^ mice, while these bands were invisible in those of Crb2^fl/fl^pod-Cre^Tg/+^ mice at 2 months of age. The band of β-actin was similar among the three groups. (**d**) Synpo, a podocyte marker, was co-localized with Crb2 in Crb2^+/+^pod-Cre^Tg/+^ or Crb2^fl/fl^ mice. No co-localization of Crb2 and Synpo was observed in Crb2^fl/fl^pod-Cre^Tg/+^ mice. Scale bars 20 μm.

Crb2 floxed heterozygous mice were generated by inserting loxP sites in introns 6 and 8 to skip exon 7 and 8 of the Crb2 gene (Fig. 1a). Podocyte-specific Crb2 knockout mice were then generated by breeding Crb2 floxed heterozygous mice with NPHS2-cre mice, also called pod-Cre^Tg/+^ transgenic (Tg) mice. These NPHS2-cre mice produce Cre recombinase in podocytes. The polymerase chain reaction (PCR) analysis disclosed a 102-bp product using the pod-Cre primers in pod-Cre mice and Crb2 flox/flox (Crb2^fl/fl^) and pod-Cre mice (Fig. 1b). Full-length gels are presented in Supplementary Fig. S1 online. The PCR analysis showed a 353-bp product using Crb2 primers in Crb2^fl/fl^ mice and Crb2^fl/fl^pod-Cre^Tg/+^ mice, and a 153-bp product in Crb2^+/+^pod-Cre^Tg/+^ mice. A Western blot analysis of isolated glomeruli using CRB2 antibody showed a strong band between 140 and 180 kDa and a weak band between 180 and 245 kDa in the glomeruli from Crb2^+/+^pod-Cre^Tg/+^ or Crb2^fl/fl^ mice. These bands were not detected in Crb2^fl/fl^pod-Cre^Tg/+^ mice (Fig. 1c). β-actin served as a loading control (Fig. 1c). Full-length blots are presented in Supplementary Fig. S2 online.

### Immunofluorescence study results are compatible with podocyte-specific Crb2 knockout in Crb2^fl/fl^pod-Cre^Tg/+^ mice

Dual staining with CRB2 and SYNPO, a podocyte marker, in normal human paraffin kidney sections showed co-localization of CRB2 and SYNPO (Supplementary Fig. S3). Dual staining with Crb2 and Synpo in paraffin kidney sections from 2-month aged mice showed co-localization of Crb2 and Synpo in Crb2^+/+^pod-Cre^Tg/+^ or Crb2^fl/fl^ mice. Co-localization of Crb2 and Synpo was not observed in Crb2^fl/fl^pod-Cre^Tg/+^ mice (Fig. 1d).

### Podocyte-specific Crb2 knockout mice develop massive albuminuria and microhematuria at 2 months of age

**Figure 2:**
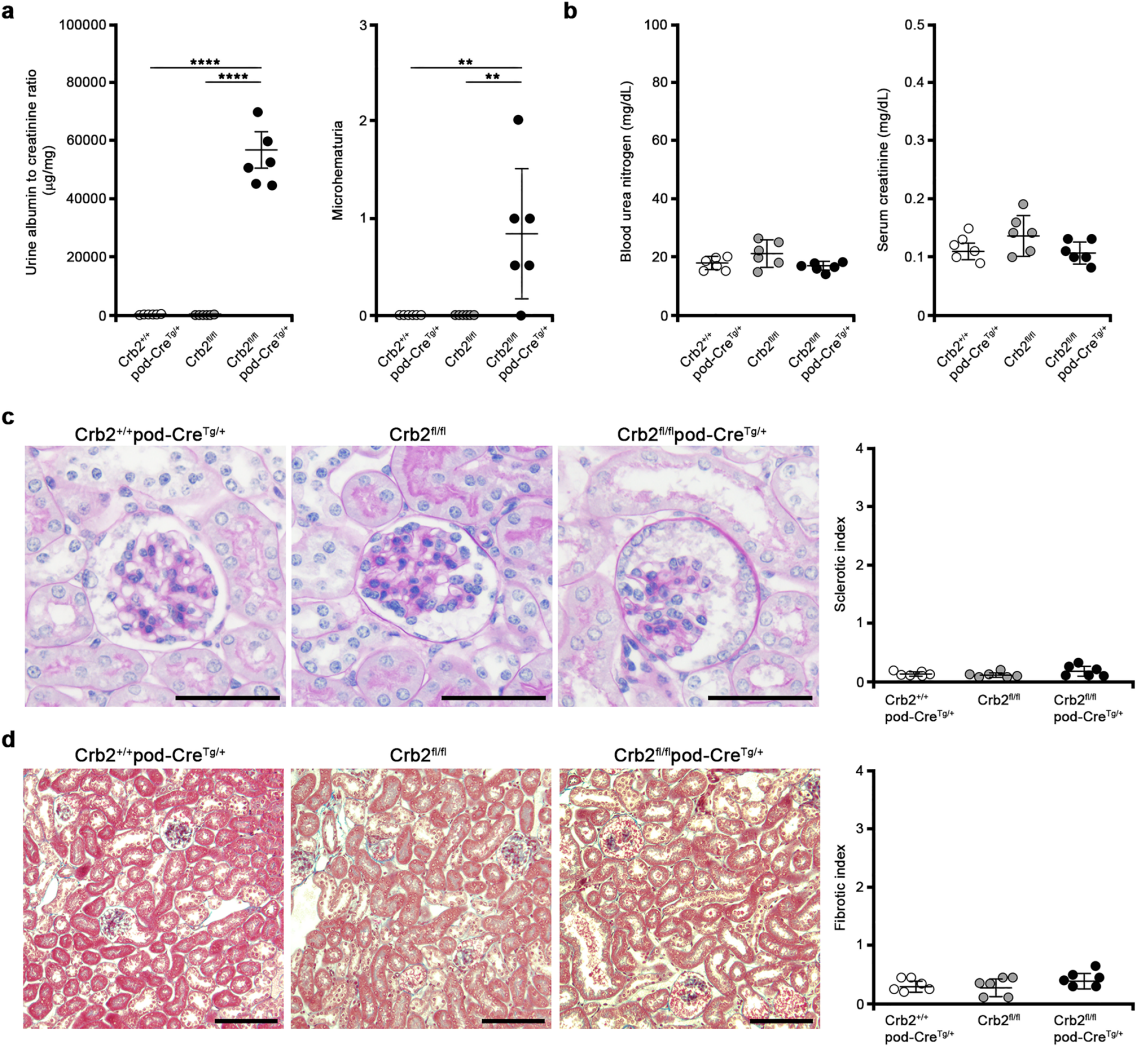
Podocyte-specific Crb2 knockout mice develop massive albuminuria and microhematuria at 2 months of age. (**a**) Massive albuminuria was observed at 2 months of age in Crb2^fl/fl^pod-Cre^Tg/+^ mice compared to Crb2^+/+^pod-Cre^Tg/+^ or Crb2^fl/fl^ mice. Microhematuria was observed at 2 months of age in Crb2^fl/fl^pod-Cre^Tg/+^ mice compared to Crb2^+/+^pod-Cre^Tg/+^ or Crb2^fl/fl^ mice. (**b**) The blood urea nitrogen and serum creatinine levels were comparable among the three groups at 2 months of age. (**c**) The sclerotic indices were comparable among the three groups at 2 months of age. Scale bars 50 μm. (**d**) The fibrotic indices were comparable among the three groups at 2 months of age. Scale bars 100 μm. Data are presented as mean ± SD. Statistical analysis by one-way analysis of variance, followed by Scheffe's multiple comparison test; *n* = 6 in each group. **P < 0.01, ****P < 0.0001.

The urine albumin-to-creatinine ratio (ACR) was significantly higher in Crb2^fl/fl^pod-Cre^Tg/+^ mice than in Crb2^+/+^pod-Cre^Tg/+^ or Crb2^fl/fl^ mice at 2 months of age (53,630 ± 9633 vs. 55 ± 51 or 46 ± 35 μg/mg·Cr, *P* < 0.0001) (Fig. 2a). Microhematuria was more recognizable in Crb2^fl/fl^pod-Cre^Tg/+^ mice than in Crb2^+/+^pod-Cre^Tg/+^ or Crb2^fl/fl^ mice (0.83 ± 0.68 vs. 0 ± 0 or 0 ± 0, *P* = 0.0083) (Fig. 2a). The blood urea nitrogen (BUN) and serum creatinine (Cr) levels were comparable among the three groups at 2 months of age (BUN; 18.1 ± 2.3, 21.4 ± 4.3, 17.0 ± 1.4 mg/dl, Cr; 0.11 ± 0.02, 0.14 ± 0.03, 0.11 ± 0.02 mg/dl) (Fig. 2b). The sclerotic (0.11 ± 0.05, 0.10 ± 0.04, 0.17 ± 0.09; Fig. 2c) and fibrotic (0.33 ± 0.11, 0.30 ± 0.16, 0.43 ± 0.13; Fig. 2d) indices were comparable among the three groups at 2 months of age.

### Podocyte-specific Crb2 knockout mice develop FSGS at 6 months of age

**Figure 3:**
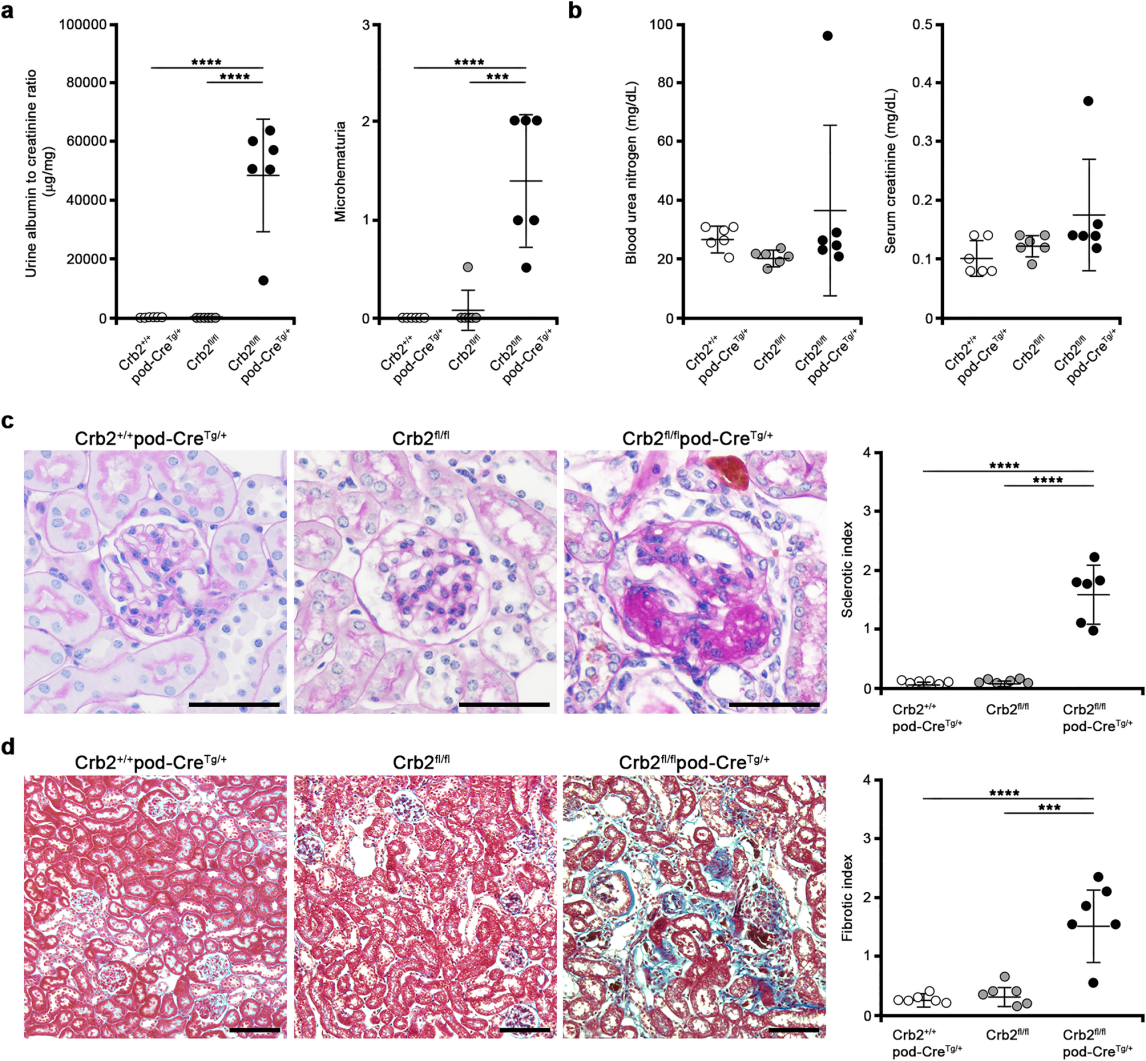
Podocyte-specific Crb2 knockout mice develop FSGS at 6 months of age. (**a**) Massive albuminuria was observed at 6 months of age in Crb2^fl/fl^pod-Cre^Tg/+^ mice compared to Crb2^+/+^pod-Cre^Tg/+^ or Crb2^fl/fl^ mice. Microhematuria was observed at 6 months of age in Crb2^fl/fl^pod-Cre^Tg/+^ mice compared to Crb2^+/+^pod-Cre^Tg/+^ or Crb2^fl/fl^ mice. (**b**) The blood urea nitrogen and serum creatinine levels were comparable among the three groups at 6 months of age. (**c**) The sclerotic indices in Crb2^fl/fl^pod-Cre^Tg/+^ mice were significantly higher than those in Crb2^+/+^pod-Cre^Tg/+^ or Crb2^fl/fl^ mice. Scale bars 50 μm. (**d**) The fibrotic indices in Crb2^fl/fl^pod-Cre^Tg/+^ mice were significantly higher than those in Crb2^+/+^pod-Cre^Tg/+^ or Crb2^fl/fl^ mice. Scale bars 100 μm. Data are presented as mean ± SD. Statistical analysis by one-way analysis of variance, followed by Scheffe's multiple comparison test; *n* = 6 in each group. ***P < 0.001, ****P < 0.0001.

Although there was no apparent albuminuria in Crb2^+/+^pod-Cre^Tg/+^ or Crb2^fl/fl^ mice, massive albuminuria was observed in Crb2^fl/fl^pod-Cre^Tg/+^ mice at 6 months of age (36 ± 4 or 56 ± 55 vs. 49,210 ± 18,594 μg/mg·Cr, *P* < 0.0001) (Fig. 3a). Microhematuria was also more recognizable in Crb2^fl/fl^pod-Cre^Tg/+^ mice than in Crb2^+/+^pod-Cre^Tg/+^ or Crb2^fl/fl^ mice (1.42 ± 0.66 vs. 0 ± 0 or 0.08 ± 0.2, *P* < 0.0001 or *P* = 0.002) (Fig. 3a). The BUN and serum creatinine levels were comparable among the three groups of mice at 6 months of age (BUN; 27.3 ± 4.1, 20.7 ± 2.4, 36.7 ± 29.3 mg/dl, Cr; 0.10 ± 0.03, 0.12 ± 0.02, 0.18 ± 0.09 mg/dl) (Fig. 3b). The sclerotic indices at 6 months of age in Crb2^fl/fl^pod-Cre^Tg/+^ mice were significantly higher than in Crb2^+/+^pod-Cre^Tg/+^ or Crb2^fl/fl^ mice (1.61 ± 0.48 vs. 0.07 ± 0.04 or 0.08 ± 0.04, *P* < 0.0001) (Fig. 3c). The fibrotic indices at 6 months of age in Crb2^fl/fl^pod-Cre^Tg/+^ mice were significantly higher than in Crb2^+/+^pod-Cre^Tg/+^ or Crb2^fl/fl^ mice (1.66 ± 0.63 vs. 0.28 ± 0.07 or 0.36 ± 0.18, *P* < 0.0001 or *P* = 0.0001) (Fig. 3d).

### Hemosiderin accumulation and CD68-positive cells are detected in the interstitium of podocyte-specific Crb2 knockout mice at 6 months of age

**Figure 4:**
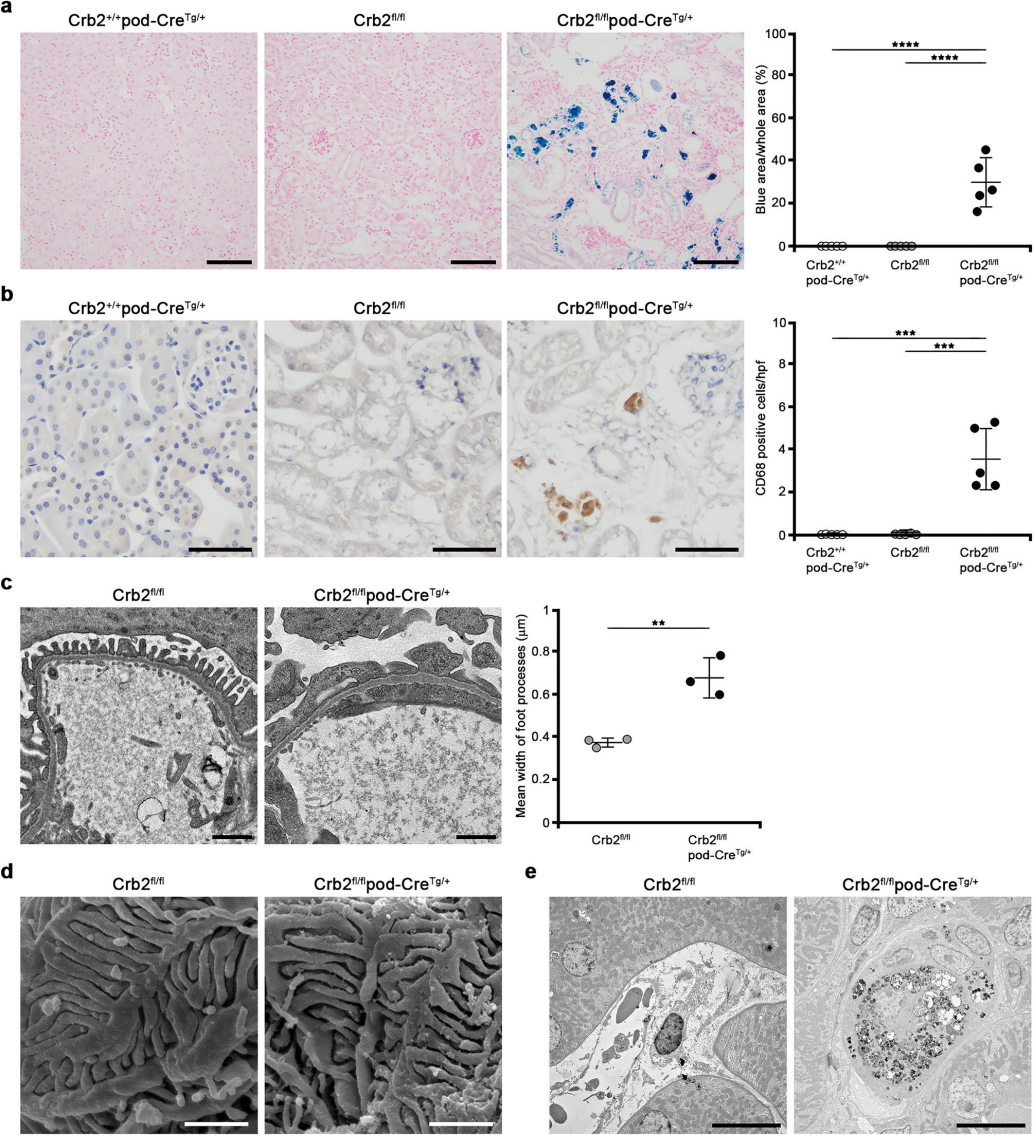
Hemosiderin accumulation and CD68-positive cells in the interstitium of podocyte-specific Crb2 knockout mice at 6 months of age and electron microscopic study. (**a**) The blue area was significantly larger in Crb2^fl/fl^pod-Cre^Tg/+^ mice than in Crb2^+/+^pod-Cre^Tg/+^ or Crb2^fl/fl^ mice. Scale bars 100 μm. (**b**) The brown cells in the interstitium of Crb2^fl/fl^pod-Cre^Tg/+^ mice were positive for CD68, suggesting that they were macrophages. CD68-positive cells were significantly more frequent in the interstitium of Crb2^fl/fl^pod-Cre^Tg/+^ mice than in that of Crb2^+/+^pod-Cre^Tg/+^ or Crb2^fl/fl^ mice. *HPF* high-power field. Scale bars 50 μm. Data are presented as mean ± SD. Statistical analysis by one-way analysis of variance, followed by Scheffe's multiple comparison test; *n* = 5 in each group. ***P < 0.001, ****P < 0.0001. (**c**) Transmission electron microscopy showed foot process effacement in Crb2^fl/fl^pod-Cre^Tg/+^ mice compared to Crb2^fl/fl^ mice. Scale bars 1 μm. The mean width of the foot processes in Crb2^fl/fl^pod-Cre^Tg/+^ mice was significantly greater than that in Crb2^fl/fl^ mice. Data are presented as mean ± SD. Statistical analysis by Student's *t*-test; *n* = 3 in each group. **P < 0.01. (**d**) Scanning electron microscopy showed damage to the foot processes' urinary sides in Crb2^fl/fl^pod-Cre^Tg/+^ mice compared to Crb2^fl/fl^ mice. Scale bars 1 μm. (**e**) Hemosiderin-laden macrophages were observed in the interstitial area of Crb2^fl/fl^pod-Cre^Tg/+^ mice, but not in that of Crb2^fl/fl^ mice, at 6 months of age. Scale bars 10 μm.

We detected brown cells in the interstitium of Crb2^fl/fl^pod-Cre^Tg/+^ mice after staining with periodic acid-Schiff (Fig. 3c), therefore we performed Berlin blue staining. The blue area was significantly larger in Crb2^fl/fl^pod-Cre^Tg/+^ mice than in Crb2^+/+^pod-Cre^Tg/+^ or Crb2^fl/fl^ mice (29.8% ± 11.5% vs. 0% ± 0% or 0% ± 0%, *P* < 0.0001), suggesting hemosiderin accumulation in the tubulointerstitial area (Fig. 4a). The brown cells in the tubulointerstitial area of Crb2^fl/fl^pod-Cre^Tg/+^ mice were positive for CD68, suggesting they were hemosiderin-laden macrophages. CD68-positive cells were significantly more frequent in the tubulointerstitial area of Crb2^fl/fl^pod-Cre^Tg/+^ mice than in Crb2^+/+^pod-Cre^Tg/+^ or Crb2^fl/fl^ mice (3.56 ± 1.48 vs. 0.02 ± 0.04 or 0.08 ± 0.13 cells per high-power field, *P* = 0.0001) (Fig. 4b).

### Electron microscopy shows morphological changes in the podocyte foot processes of podocyte-specific Crb2 knockout mice at 6 months of age

Transmission electron microscopy revealed increased segmental foot process effacement in Crb2^fl/fl^pod-Cre^Tg/+^ mice compared to Crb2^fl/fl^ mice at 6 months of age (Fig. 4c). The mean width of the foot processes in Crb2^fl/fl^pod-Cre^Tg/+^ mice was significantly greater than in Crb2^fl/fl^ mice (0.683 ± 0.021 vs. 0.377 ± 0.095 μm, *P* = 0.0054) (Fig. 4c). Scanning electron microscopy revealed damage to the podocyte foot process's urinary side in Crb2^fl/fl^pod-Cre^Tg/+^ mice compared to Crb2^fl/fl^ mice at 6 months of age (Fig. 4d). Hemosiderin-laden macrophages were observed in the interstitial area of Crb2^fl/fl^pod-Cre^Tg/+^ mice but not in that of Crb2^fl/fl^ mice at 6 months of age (Fig. 4e).

### RNA sequencing (RNA-seq) analysis

We performed RNA-seq of the total kidney RNA extracts from Crb2^fl/fl^pod-Cre^Tg/+^ and Crb2^fl/fl^ mice at 6 months of age. The fragments per kilobase of exon per million reads mapped (FPKM) of 23,913 genes are described in Supplementary Table S1. After removing the zero mapped reads, the log 2 ratio was calculated from the 13,880 genes (Supplementary Table S2). There were 3174 and 666 significant genes before and after correction with the Benjamini and Hochberg method (Supplementary Fig. S4, S5a, Supplementary Table S2). Among 666 significant genes, 477 genes were upregulated > 2-fold, and 16 genes were downregulated > 2-fold in Crb2^fl/fl^pod-Cre^Tg/+^ mice compared to Crb2^fl/fl^ mice (Supplementary Table S2). As podocyte-related genes were not included in the 477 upregulated and 15 downregulated genes, 3174 genes before correction with the Benjamini and Hochberg method were reviewed. There were 1173 > 2-fold upregulated genes and 91 > 2-fold downregulated genes in Crb2^fl/fl^pod-Cre^Tg/+^ mice compared to Crb2^fl/fl^ mice (Supplementary Table S2). We listed the top 50 upregulated (Supplementary Table S3) and downregulated (Supplementary Table S4) genes. The slit diaphragm-associated protein Nphs2 was included in the top 50 downregulated genes, with a significant difference in expression (log 2 ratio: − 1.46, *P* = 0.0478). Other slit diaphragm-associated proteins including Cd2ap (log 2 ratio: 0.52, *P* = 0.059), Neph1 (no data), Nphs1 (log 2 ratio: − 0.93, *P* = 0.05), Synpo (log 2 ratio: 0.40, *P* = 0.403), and Tjp1 (log 2 ratio: 0.28, *P* = 0.262) showed no significant changes. Regarding apical membrane proteins, Podxl was significantly downregulated (log 2 ratio: − 0.75, *P* = 0.026), and Pdpn was significantly upregulated (log 2 ratio: 1.8, *P* = 0.008).

### The protein expressions of Nphs2, Podxl, and Nphs1 and the number of Wt1-positive cells in the glomeruli are decreased in podocyte-specific Crb2 knockout mice at 6 months of age

**Figure 5:**
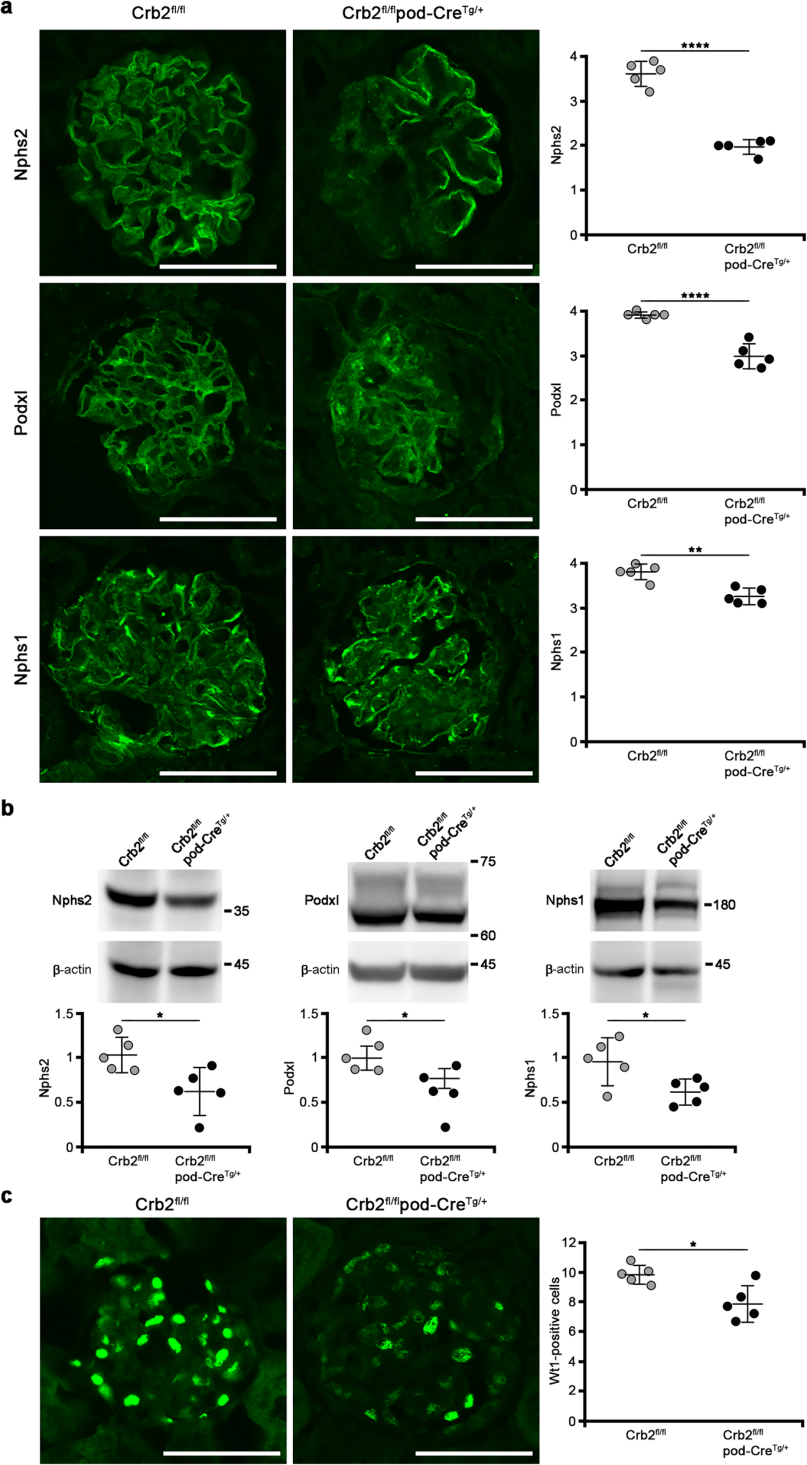
The protein expressions of Nphs2, Podxl, and Nphs1 and the number of Wt1 positive cells in the glomeruli were decreased in podocyte-specific Crb2 knockout mice. (**a**) An immunofluorescence study showed that Nphs2, Podxl, and Nphs1 expression significantly decreased in Crb2^fl/fl^pod-Cre^Tg/+^ mice compared to Crb2^fl/fl^ mice. Scale bars 50 μm. (**b**) The protein expressions of Nphs2, Podxl, and Nphs1 were significantly decreased in Crb2^fl/fl^pod-Cre^Tg/+^ mice compared to Crb2^fl/fl^ mice. (**c**) Wt1-positive cells were significantly decreased in the glomeruli of Crb2^fl/fl^pod-Cre^Tg/+^ mice compared to those of Crb2^fl/fl^ mice. Data are presented as mean ± SD. Statistical analysis by Student's *t*-test; *n* = 5 in each group. *P < 0.05, **P < 0.01, ****P < 0.0001.

We examined whether the expression of Nphs2, Podxl, or Nphs1 is decreased in the glomeruli. An immunofluorescence study showed that the expressions of Nphs2 (1.98 ± 0.16 vs. 3.62 ± 0.28, *P* < 0.0001), Podxl (2.98 ± 0.28 vs. 3.9 ± 0.07, *P* < 0.0001), and Nphs1 (3.26 ± 0.18 vs. 3.80 ± 0.19, *P* = 0.0017) were significantly decreased in Crb2^fl/fl^pod-Cre^Tg/+^ mice compared to Crb2^fl/fl^ mice (Fig. 5a). The protein expressions of Nphs2 (0.627 ± 0.259 vs. 1.039 ± 0.191, *P* = 0.0211), Podxl (0.775 ± 0.110 vs. 1.003 ± 0.135, *P* = 0.0189), and Nphs1 (0.625 ± 0.139 vs. 0.967 ± 0.260, *P* = 0.0320) were significantly decreased in Crb2^fl/fl^pod-Cre^Tg/+^ mice compared to Crb2^fl/fl^ mice (Fig. 5b). Full-length blots are presented in Supplementary Fig. S6, S7 online. Wt1-positive cells (7.9 ± 1.2 vs. 9.8 ± 0.7, *P* = 0.0131) were significantly decreased in the glomeruli of Crb2^fl/fl^pod-Cre^Tg/+^ mice compared to those of Crb2^fl/fl^ mice (Fig. 5c).

### Circulating permeability factors in mouse serum samples at 6 months of age

**Figure 6:**
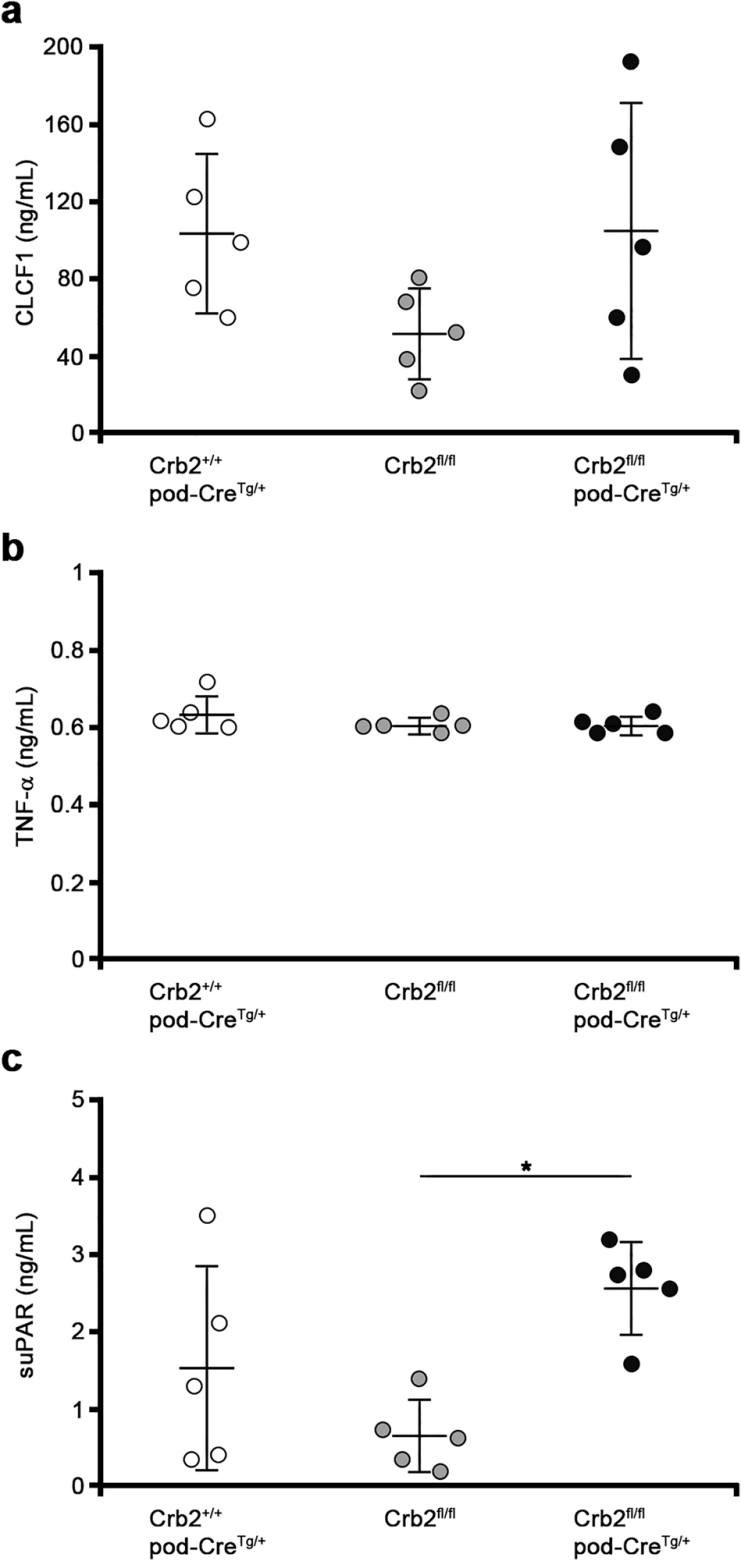
The circulating permeability factors in the mouse serum samples at 6 months of age. (**a**) The serum levels of CLCF1 were comparable among the three groups. (**b**) The serum levels of TNF-α were comparable among the three groups. (**c**) The serum levels of suPAR were significantly elevated in the Crb2^fl/fl^pod-Cre^Tg/+^ mice compared to the Crb2^fl/fl^ mice while there was no significant difference between serum levels of the Crb2^fl/fl^pod-Cre^Tg/+^ mice and the Crb2^+/+^pod-Cre^Tg/+^ mice. Data are presented as the mean ± SD. The statistical analysis was performed by a one-way analysis of variance, followed by Scheffe's multiple comparison test; *n* = 5 in each group. *P < 0.05. *CLCF1* cardiotrophin like cytokine factor 1, *TNF-α* tumor necrosis factor-α, *suPAR* soluble urokinase-type plasminogen activator receptor.

The serum levels of either cardiotrophin like cytokine factor 1 (CLCF1) or tumor necrosis factor (TNF)-α were found to be comparable among the three groups (Fig. 6a,b). The serum levels of soluble urokinase-type plasminogen activator receptor (suPAR) were significantly elevated in the Crb2^fl/fl^pod-Cre^Tg/+^ mice compared to the Crb2^fl/fl^ mice (2.56 ± 0.60 vs. 0.65 ± 0.46, *P* = 0.016) while there was no significant difference between the serum levels of the Crb2^fl/fl^pod-Cre^Tg/+^ mice and the Crb2^+/+^pod-Cre^Tg/+^ mice (Fig. 6c).

### Generation of CRB2 knockout human immortalized podocytes

**Figure 7:**
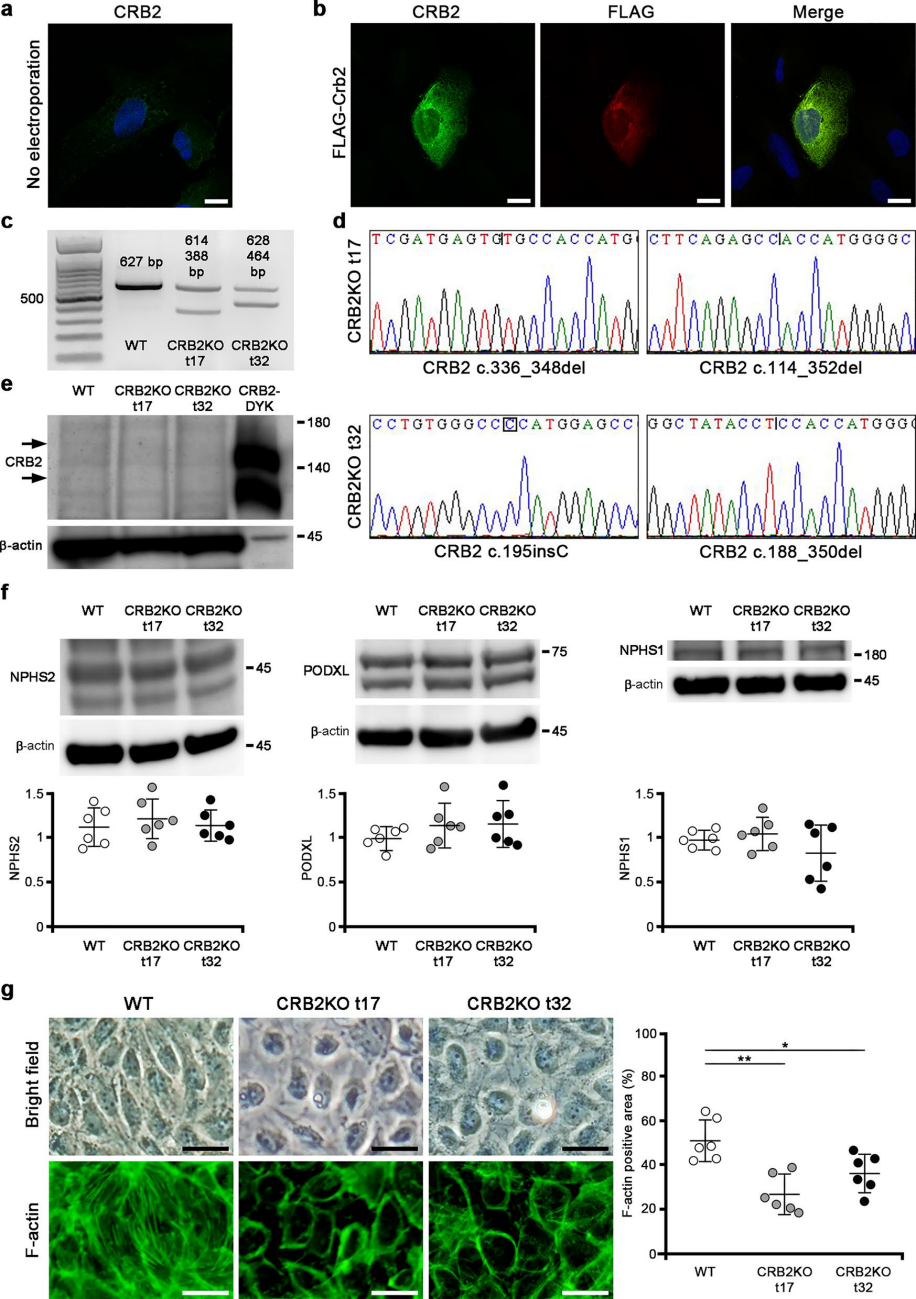
Generation of CRB2 knockout human immortalized podocytes. (**a**) The endogenous expression of CRB2 in differentiated human culture podocytes was weakly observed in the nucleus and cytoplasm. Scale bar 20 μm. (**b**) In differentiated human podocytes electroporated with carboxy-terminal FLAG-tagged mouse full-length Crb2, the staining with rabbit anti-CRB2 antibody and mouse anti-FLAG antibody was co-localized in the cytoplasm and was weak in the plasma membrane. Scale bars 20 μm. (**c**) While there was a 627-bp product in wild-type podocytes, there were 614- and 388-bp products in CRB2 knockout t17 clone and 628- and 464-bp products in CRB2 knockout t32 clone. The left lane was the 100-bp marker. (**d**) The CRB2 knockout t17 clone possessed mutations in an allele with c.336_348del TGCATCCCGGCCG, which caused a frameshift mutation and premature stop codon due to a 13-bp deletion, and in the other allele with c.114–352del, which caused a frameshift mutation and premature stop codon due to a 239-bp deletion. The CRB2 knockout t32 clone possessed mutations in an allele with c.195insC, which caused a frameshift mutation and premature stop codon due to a 1-bp insertion, and in the other allele with c.188–350del, which caused a frameshift mutation and premature stop codon due to a 163-bp deletion. (**e**) There were weak bands above and below 140 kDa marker with CRB2 antibody in the lysates of wild-type podocytes, while these bands were invisible in those of CRB2 knockout t17 and t32 clones. The positive control was a lysate from human embryonic kidney 293 cells transfected with CRB2-DYK. β-actin served as a loading control. (**f**) The protein expressions of NPHS2, PODXL, and NPHS1 were comparable among wild-type, CRB2 knockout t17, and t32 clones. (**g**) The F-actin positive area in CRB2 knockout t17 or t32 clones significantly decreased compared to wild-type human podocytes. Scale bars 50 μm. Data are presented as mean ± SD. Statistical analysis by one-way analysis of variance, followed by Scheffe's multiple comparison test; *n* = 6 in each group. *P < 0.05, **P < 0.01. *CRB2KO* CRB2 knockout, *WT* wild-type.

The endogenous expression of CRB2 in differentiated human culture podocytes was weakly observed in the nucleus and cytoplasm (Fig. 7a). In differentiated human podocytes electroporated with carboxy-terminal FLAG-tagged mouse full-length Crb2, the staining of CRB2 and FLAG co-localized in the cytoplasm, although their staining was weak in the plasma membrane (Fig. 7b). We used four different single-guide RNA (sgRNA)s targeting exon 2 of CRB2 (sgRNA1, sgRNA2484, sgRNA2491, sgRNA 2497) to generate CRB2 knockout human podocytes. Single-cell cloning was performed in a 96-well plate for each sgRNA. The sequence analysis of 17 clones (t1–t17) from sgRNA1, 2 (t18–t19) from sgRNA2484, 17 (t20–t36) from sgRNA2491, and 6 (t37–t42) from sgRNA2497 revealed 5 candidate clones (t9, t17, t21, t29, t32) containing indel mutations. Of the five clones, t17 and t32 were confirmed as a CRB2 knockout clone after repeated sequence analyses. We detected a 627-bp product in wild-type podocytes, 614-bp and 388-bp products in CRB2 knockout t17 clone, and 628-bp and 464-bp products in the CRB2 knockout t32 clone (Fig. 7c). Full-length gels are presented in Supplementary Fig. S8 online. The CRB2 knockout t17 clone contained a mutation in an allele (c.336_348del TGCATCCCGGCCG) that caused a frameshift mutation and premature stop codon due to a 13-bp deletion (mutant protein with 136 amino acids) and a mutation in the other allele (c.114_352del) that caused a frameshift mutation and premature stop codon due to a 239-bp deletion (mutant protein of 200 amino acids) (Fig. 7d). The CRB2 knockout t32 clone contained a mutation in an allele (c.195insC) that results in a mutant protein with 110 amino acids and a mutation in the other allele (c.188_350del) that results in a mutant protein of 86 amino acids due to a 163-bp deletion (Fig. 7d). A Western blot analysis with CRB2 antibody showed weak bands above and below 140 kDa, which were almost of similar sizes to the CRB2-DYK positive control. However, these bands were invisible in the CRB2 knockout t17 and t32 clones (Fig. 7e). β-actin served as a loading control (Fig. 7e). Full-length blots are presented in Supplementary Fig. S9 online. The protein expressions of NPHS2, PODXL, and NPHS1 were comparable among the wild-type, CRB2 knockout t17 and t32 clones (Fig. 7f). Full-length blots are presented in Supplementary Fig. S10, S11 online. MPP5 (PALS1) and PATJ expressions were also comparable between wild-type podocytes and CRB2 knockout clones (Supplementary Fig. S12). Full-length blots are presented in Supplementary Fig. S13 online. The morphology of CRB2 knockout human podocytes was rounded compared to wild-type human podocytes. In an attempt to find an explanation, we evaluated the expression of F-actin. The F-actin positive area was significantly decreased in CRB2 knockout t17 (*P* = 0.0011) or t32 (*P* = 0.0359) clones compared to wild-type human podocytes (Fig. 7g).

### Apoptosis and adhesion assay

**Figure 8:**
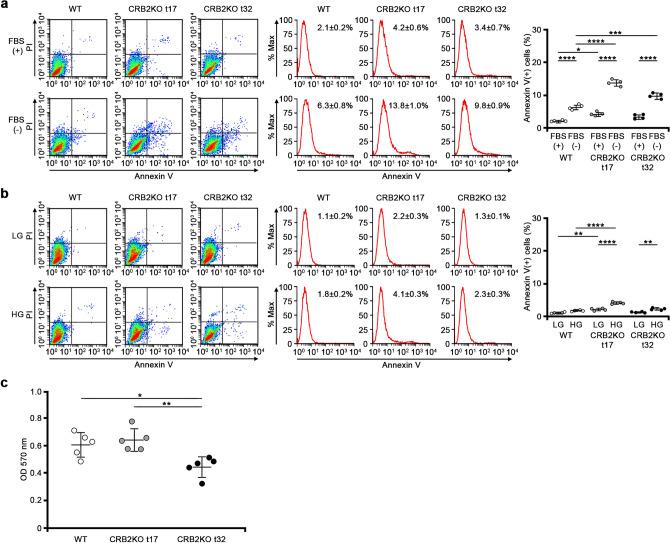
Apoptosis and adhesion assay. (**a**) Apoptotic assay demonstrated that CRB2 knockout t17 and t32 clones were more susceptible to apoptosis under serum starvation than wild-type human podocytes. (**b**) CRB2 knockout t17 clone was more susceptible to apoptosis under high glucose conditions than wild-type human podocytes, while there was no significant difference between CRB2 knockout t32 clone and wild-type podocytes. Data are presented as mean ± SD. Statistical analysis by one-way analysis of variance, followed by Scheffe's multiple comparison test; *n* = 4 in each group. *P < 0.05, **P < 0.01, ***P < 0.001, ****P < 0.0001. (**c**) Adhesion assay showed decreased adhesive ability in CRB2 knockout t32 clone compared to wild-type podocytes or CRB2 knockout t17 clone while there was no significant difference between CRB2 knockout t17 and wild-type podocytes. Data are presented as mean ± SD. Statistical analysis by one-way analysis of variance, followed by Scheffe's multiple comparison test; *n* = 5 in each group. *P < 0.05, **P < 0.01. *CRB2KO* CRB2 knockout, *FBS* fetal bovine serum, *HG* high glucose, *LG* low glucose, *WT* wild-type.

The apoptosis assay demonstrated that CRB2 knockout t17 (*P* < 0.0001) and t32 (*P* = 0.0002) clones are more susceptible to apoptosis under serum starvation compared to wild-type human podocytes (Fig. 8a). CRB2 knockout t17 clone was more susceptible to apoptosis under high glucose than wild-type human podocytes (*P* < 0.0001). However, there was no significant difference between CRB2 knockout t32 clone and wild-type podocytes (Fig. 8b). Adhesion assay showed decreased adhesive ability in CRB2 knockout t32 clone compared to wild-type podocytes or CRB2 knockout t17 clone. There was no significant difference between CRB2 knockout t17 and wild-type podocytes (Fig. 8c).

## Discussion

The present study showed that podocyte-specific Crb2 knockout mice developed massive albuminuria and microhematuria at 2 months of age, FSGS with tubulointerstitial fibrosis at 6 months of age, and that podocyte-specific deficiency of CRB2 was associated with segmental foot process effacement, significant downregulation of slit diaphragm-associated proteins (Nphs1, Nphs2), apical membrane protein (Podxl) and decreased number of glomerular Wt1-positive cells compared to their negative control counterparts. In addition, this study demonstrated that CRB2 knockout human immortalized podocytes showed abnormal morphology, decreased expression and distribution of F-actin, and high susceptibility to apoptosis compared to wild-type podocytes. Overall, these observations underscore the critical role of CRB2 in the glomerular filtration barrier.

Disruption of the Crb2 protein in podocytes might cause actin cytoskeleton reorganization in podocytes' foot process. The 37-amino-acid cytoplasmic domain of CRB2 is 100% identical in humans and mice. This cytoplasmic domain contains a binding site for the FERM domain of moesin that binds to actin filaments through its carboxy-terminal domain^12^. In our present knockout mouse model, the deletion of exons 7 and 8 caused a frameshift mutation, resulting in a mutant Crb2 protein of 370 amino acids lacking the cytoplasmic domain. This mutant Crb2 protein without the FERM binding motif in podocytes is likely unable to bind to moesin, and consequently, reorganization of the actin cytoskeleton occurs. The differential gene expression pattern in podocyte-specific Crb2 knockout mice shown by the RNA-seq study (Supplementary Fig. S5b), the abnormal rounded morphology, and the decreased expression and cellular distribution of F-actin in CRB2 knockout human immortalized podocytes compared to their wild-type counterparts support the hypothesis that the actin cytoskeleton is abnormally reorganized in podocyte-specific Crb2-deficient mice. Interestingly, CRB2 knockout human immortalized podocytes were more susceptible to apoptosis under serum starvation than wild-type human podocytes. Based on these observations, we speculate that loss-of-function of CRB2 in podocytes induces abnormal reorganization of the actin cytoskeleton and major susceptibility to apoptosis, leading to increased loss of podocytes and ultimately to focal segmental glomerulosclerosis.

The Nphs2 expression was significantly downregulated in podocyte-specific Crb2 knockout mice before correcting with the Benjamini and Hochberg method. However, the expression of other slit diaphragm-associated proteins was comparable. We then assessed the protein expressions of Nphs2 and Nphs1 and found that they were significantly decreased in podocyte-specific Crb2 knockout mice. The slit diaphragm-associated protein NPHS2 is a hairpin-like membrane protein with intracellular amino- and carboxy-terminal domains^19^. The carboxy-terminal domain of NPHS2 interacts with NPHS1 and CD2AP^20^. This CD2AP is an adapter protein that directly interacts with the actin cytoskeleton through its carboxy-terminal domain^21^. Nphs2 knockout mice develop massive proteinuria and die 5 weeks after birth^22^. The decreased expression of Nphs1 and Nphs2 was associated with a decreased number of Wt1-positive cells in the glomeruli of podocyte-specific Crb2 knockout mice, further suggesting that an accelerated loss of podocytes occurred in our current disease mouse model.

The RNA-seq results before correction by the Benjamini and Hochberg method revealed that the Podxl expression was significantly downregulated in podocyte-specific Crb2 knockout mice. The protein expression of Podxl was also significantly decreased in the mouse model. PODXL is a major polyanion sialoprotein of podocytes localized on podocytes' apical surfaces, as demonstrated by immunoelectron microscopy^23^. Podxl knockout mice are anuric and die within 24 h of birth^24^. PODXL interacts with the actin cytoskeleton through Ezrin and Nherf2^25,26^, and this interaction is disrupted in rats with PAN nephritis^26^. These observations suggest that the decreased expression of Podxl also contributes to the reorganization of the actin cytoskeleton in podocytes, which is manifested as a foot process effacement in our present podocyte-specific Crb2 knockout mice.

Microhematuria is a common observation during the progression of chronic kidney disease. Therefore, microhematuria and albuminuria observed in our podocyte-specific Crb2 knockout mice are probably indicators of progressive tubulointerstitial fibrosis^27^. Our present mouse model also showed the presence of CD68-positive and hemosiderin-containing cells in the vicinity of proximal renal tubules. The uptake of hemoglobin by proximal tubular epithelial cells may cause oxidative injury^28^. Indeed, the KEGG pathways in a David functional analysis of 1173 > 2-fold upregulated genes showed that many genes were associated with the complement system (Supplementary Table S5).

We measured the circulating factors, such as CLCF1, suPAR, and TNF-α^29–32^. In line with the primary causes of FSGS through the deletion of Crb2 in podocytes, the circulating FSGS factors were largely found to be normal.

Several other mechanisms might be involved in the development of FSGS in the podocyte-specific Crb2 knockout mice. A gain-and-loss of function study for Crumbs in Drosophila revealed dysregulation in the Hippo signaling pathway through its FERM domain-binding motif^33,34^. The role of CRB2 in podocyte development through tyrosine phosphorylation was associated with the activation of mammalian target of rapamycin complex 1^35^. Although the complete-knockout of Crb2 is associated with embryonic lethality due to defects in epithelial cell polarization^16^, our present mouse model showed no embryonic lethality. This discrepancy may be explained by the use of Cre recombinase under the control of the NPHS2 promoter in the present model. Therefore, the present model may depict the role of Crb2 in the structural integrity of podocytes rather than its role in cell polarity.

Several limitations associated with the present study warrant mention. First, the phenotype of podocyte-specific Crb2 knockout mice was much milder than changes observed in patients with CRB2 mutations. In mice, the complete Crb2 knockout mice showed embryonic lethality, and in humans, intrauterine growth restriction has been reported. Therefore, the present podocyte-specific deletion of Crb2 might be more specific to the kidney phenotype. Although we examined urine at 1 month of age in four mice as a preliminary experiment and only the Crb2^fl/fl^pod-Cre^Tg/+^ mouse showed massive albuminuria (Supplementary Table S6), we have not conducted such examinations in younger mice to clarify the timing of the onset of albuminuria. Second, the fact that we performed an analysis of the RNA-seq using whole kidney tissues instead of using isolated glomeruli to enrich for podocytes or conducting a single-cell analysis with podocytes to show changes in slit diaphragm genes was another limitation of our present study. This may explain why we failed to detect podocyte-related genes after correction with the Benjamini and Hochberg method. Third, although the numbers of glomerular WT1-positive cells were decreased in the present mouse model, there was a possibility that the WT1 expression might be downregulated in podocytopathies, even where the podocyte number remained intact. Fourth, the endogenous expression of CRB2 in immortalized human podocytes was much weaker compared to mouse model and might not reflect the real situation in in vivo model. A previous article also reported that the endogenous CRB2 expression in the mouse immortalized podocyte cell line was undetectable^35^.

Overall, this study's results suggest that the specific deprivation of Crb2 in podocytes causes alteration of the actin cytoskeleton, dysfunction, and accelerated apoptosis of podocytes leading ultimately to focal segmental glomerulosclerosis.

## Methods

### Animal experiments

Crb2 floxed mice were designed so that loxP sites could skip exons 7 and 8 of the Crb2 gene. NPHS2-cre mice were purchased from Jackson Laboratory (Bar Harbor, ME, USA). Podocyte-specific Crb2 knockout mice were generated by breeding Crb2^fl/fl^ mice with pod-Cre^Tg/+^ mice. All animal experiments were approved by the Animal Care and Use Committee of Mie University (No. 27-32) and Karolinska Institute (N271-13) and all methods were performed in accordance with the relevant guidelines and regulations. The reporting in the manuscript followed the recommendations in the ARRIVE guidelines.

### Urine and blood chemistry analyses

ACR, microhematuria, BUN, and Cr levels were examined at 2 or 6 months of age (*n* = 6 each). The ACR levels were measured using an immunoturbidimetric method for urinary albumin (Shibayagi, Gunma, Japan) and an enzymatic assay for urinary creatinine (Wako, Tokyo, Japan). Microhematuria was measured using dipsticks Uropaper III (Eiken Kagaku, Tokyo, Japan), and semiquantification was performed by dividing into five categories: (−) as 0, (+/−) as 0.5, (1+) as 1, (2+) as 2, and (3+) as 3. The BUN and Cr levels were measured as described previously^36^.

### Renal histology

Periodic acid-Schiff staining or Masson's trichrome staining was performed at 2 or 6 months of age (*n* = 6 each). The sclerotic index was calculated in 50 randomly selected glomeruli from each mouse, and the fibrotic index was calculated in 20 randomly selected areas from each mouse, as described previously^37^.

### Immunofluorescence study

Human kidney paraffin sections were purchased from Zyagen Laboratories (San Diego, CA, USA). Mouse paraffin sections (3 μm) were prepared from Crb2^+/+^pod-Cre^Tg/+^ mice, Crb2^fl/fl^ mice, and Crb2^fl/fl^pod-Cre^Tg/+^ mice at 2 months of age. Antigen retrieval in sodium citrate buffer (10 mM sodium citrate, 0.05% Tween 20, pH 6.0) was performed at 96 °C for 60 min. The primary and secondary antibodies were rabbit anti-CRB2 antibody (Abcam, Cambridge, UK) and mouse anti-SYNPO antibody (PROGEN Biotechnik GmbH, Heidelberg, Germany), and Alexa Fluor 488 goat anti-rabbit and 546 goat anti-mouse IgG (H&L) (ThermoFisher Scientific, Waltham, MA, USA), respectively. The nuclei were stained with 4′,6-diamindino-2-phenylindole (DAPI).

### Western blotting

The glomeruli were isolated from Crb2^+/+^pod-Cre^Tg/+^ mice, Crb2^fl/fl^ mice, and Crb2^fl/fl^pod-Cre^Tg/+^ mice at 2 months of age (*n* = 3 each) by perfusing magnetic beads without collagenase digestion^38^. The samples were separated on 4–12% Bis–Tris gels and transferred to polyvinylidene difluoride membranes. After blocking, the membranes were cut around 60 kDa and incubated with rabbit anti-CRB2 antibody (1:2000) (Abcam) and mouse anti-β-actin antibody (1:2000) (Cell Signaling, Danvers, MA, USA) at 4 °C overnight. After washing, the membranes were incubated with horseradish peroxidase-linked donkey anti-rabbit or anti-mouse antibody (1:3000) (GE Healthcare, Chicago, IL, USA). Protein detection was performed using ECL prime Western blotting detection reagent (GE Healthcare). β-actin served as a loading control.

### Berlin blue staining

Mouse paraffin sections (3 μm) from Crb2^+/+^pod-Cre^Tg/+^ mice, Crb2^fl/fl^ mice, and Crb2^fl/fl^pod-Cre^Tg/+^ mice at 6 months of age (*n* = 5 each) were stained with the Berlin blue staining set (Wako) according to the manufacturer's instructions. After washing, the sections were incubated with 0.1% Nuclear fast red-aluminum sulfate solution (Merck Millipore, Burlington, MA, USA) for 5 min at room temperature. The blue area/whole area was calculated in 10 randomly selected areas from each mouse using the ImageJ software program version 1.51 (https://imagej.nih.gov/ij/, National Institutes of Health, Bethesda, MD, USA).

### CD68 staining

Mouse paraffin kidney sections (3 μm) from Crb2^+/+^pod-Cre^Tg/+^ mice, Crb2^fl/fl^ mice, and Crb2^fl/fl^pod-Cre^Tg/+^ mice at 6 months of age were incubated with proteinase K (20 μg/ml) for 20 min at room temperature (*n* = 5 each). Staining was performed using the VECTASTAIN Elite ABC Kit (rabbit IgG) (Vector Laboratories, Burlingame, CA, USA). The primary antibody was the rabbit anti-CD68 antibody (Abcam). The sections were incubated with 1% 3,3′-Diaminobenzidine (DAB) solution and counterstained with Mayer's Hematoxylin solution. CD68-positive cells were counted in 10 randomly selected high-power fields from each mouse.

### Transmission and scanning electron microscopy

The kidney samples of Crb2^fl/fl^ mice or Crb2^fl/fl^pod-Cre^Tg/+^ mice at 6 months of age (*n* = 3 each) were pre-fixed in 0.1 M phosphate buffer with 2.5% glutaraldehyde and 2% paraformaldehyde (PFA) and post-fixed in 2% osmium tetra-oxide for 2 h at 4 °C. For transmission electron microscopy, the samples were embedded in epoxy resin, and ultrathin sections were examined with a HITACHI H-700 (Hitachi, Tokyo, Japan). The mean width of the foot processes with a glomerular basement membrane exceeding 400 μm was calculated in two to three glomeruli per mouse, as previously described^39^. For scanning electron microscopy, the samples were dehydrated, coated with an osmium plasma ion coater, and examined with an SM-7500F (JEOL, Tokyo, Japan).

### Semiquantification in immunofluorescence

Mouse frozen kidney sections (7 μm) were prepared from Crb2^fl/fl^ mice or Crb2^fl/fl^pod-Cre^Tg/+^ mice at 6 months of age (each *n* = 5). Fixation was performed in acetone at − 20 °C for 10 min. The primary and secondary antibodies were rabbit anti-NPHS2 antibody^40^ or rabbit anti-PODXL antibody^41^ or rabbit anti-NPHS1 antibody^42^ or rabbit anti-WT1 antibody (Abcam) and Alexa Fluor 488 goat anti-rabbit IgG (H&L), respectively. The images of 10 randomly selected glomeruli in each mouse were captured with an FV1000 confocal laser scanning microscope (Olympus, Tokyo, Japan). The semiquantification of the expression of Nphs2, Podxl, and Nphs1 was performed as described previously^41^. Total kidney lysates were prepared from Crb2^fl/fl^ mice or Crb2^fl/fl^pod-Cre^Tg/+^ mice at 6 months of age (each *n* = 5) for Western blotting. The membranes were cut around 60 kDa before incubation with primary antibodies. The membrane after incubation with rabbit anti-NPHS2 antibody was stripped and then reprobed with mouse anti-β-actin antibody. Wt1-positive cells were counted in 30 glomeruli per mouse.

### Measurement of the circulating permeability factors in the mouse serum samples

The serum CLCF1, suPAR, and TNF-α levels were examined at 6 months of age (*n* = 5 each) using a Mouse Cardiotrophin Like Cytokine Factor 1 ELISA Kit (ABclonal, Woburn, MA, USA), Mouse Soluble Urokinase Plasminogen Activator Receptor ELISA Kit (MyBioSource, San Diego, CA, USA), and Mouse TNF alpha ELISA Kit (Abcam) according to the manufacturer's recommendations. All experiments were performed in duplicate with the serum samples diluted at 1:10.

### Cell culture

Human immortalized podocytes were cultured in RPMI 1640 medium (Wako) with 10% fetal bovine serum, insulin-transferrin-selenium-A (Wako), and penicillin/streptomycin at 33 °C (in 5% CO_2_) to promote cell propagation^43^. The cells were moved to a 37 °C (in 5% CO_2_) incubator for 2 weeks to promote cell differentiation. Differentiated human podocytes were reseeded on type I collagen-coated cover glasses and fixed with 4% PFA for 20 min. The cover glasses were incubated with rabbit anti-CRB2 antibody (Abcam) or no primary antibody. The slides were then mounted after incubation with Alexa Fluor 488 goat anti-rabbit IgG (H&L) and DAPI. Kozak sequence (GCCACC)-full-length mouse Crb2 cDNA without a stop codon was cloned into the EcoRI and SalI sites of pFLAG-CMV-5a. The carboxy-terminal FLAG-tagged mouse full-length Crb2 or FLAG-empty vector was electroporated into differentiated human podocytes (2 × 10^6^ cells) with 4D-Nucleofector (Lonza, Basel, Switzerland). The electroporated cells were reseeded onto type I collagen-coated cover glasses and fixed in 4% PFA for 20 min. The primary and secondary antibodies were rabbit anti-CRB2 antibody (Abcam) and mouse anti-FLAG antibody (Sigma Aldrich, St. Louis, MO, USA), and Alexa Fluor 488 goat anti-rabbit and 546 goat anti-mouse IgG (H&L), respectively. The nuclei were stained with DAPI and examined with an FV1000 confocal laser scanning microscope.

### Generation of CRB2 knockout human immortalized podocytes

CRB2 knockout podocytes were generated using a CRISPR-Cas9 system. Four different single-guide RNA (sgRNA)s that targeted exon 2 of CRB2 were used. The sequences of CRB2 sgRNA1, sgRNA2484, sgRNA2491, and sgRNA2497 were 5′-ACATCGATGAGTGTGCATCC-3′, 5′-GAGAGTGGTGGCTATACCTG-3′, 5′-TGGCTATACCTGTGGGCCCA-3′, and 5′-AGAGTGGTGGCTATACCTGT-3′, respectively. Each sgRNA (1.25 μg) was used to transfect undifferentiated podocytes (0.5 × 10^5^ cells/well) at 33 °C with Lipofectamine CRISPRMAX, TrueCut Cas9 Protein v2, Cas9 plus reagent (ThermoFisher Scientific). After 3 days, single-cell cloning was performed by dilution in 96-well plates. After 3 weeks, genomic DNA was extracted from each clone, and sequence analysis was performed to detect indels. For Western blotting, cell lysates were prepared from wild-type or CRB2 knockout human podocytes, and positive control was a lysate of human embryonic kidney 293 cells transfected with pcDNA3.1-full-length human CRB2-DYK. The membranes were cut around 60 kDa before incubation with primary antibodies. The membrane after incubation with rabbit anti-NPHS2 antibody was stripped and then reprobed with mouse anti-β-actin antibody. Primary antibodies were anti-CRB2 antibody (Abcam) (1:2000), mouse anti-β-actin antibody (1:2000) (Cell Signaling), rabbit anti-NPHS2 antibody (1:1000)^37^, rabbit anti-PODXL antibody (1:1000) (Proteintech, Rosemont, IL, USA), mouse anti-NPHS1 antibody (1:1000) (Santa Cruz Biotechnology, Dallas, TX, USA), rabbit anti-PATJ antibody (1:1000) (Sigma Aldrich), and rabbit anti-MPP5 antibody (1:1000) (Sigma Aldrich). F-actin positive areas (each *n* = 6) were calculated after staining with Phalloidin-iFluor 488 (Cayman Chemical, Ann Arbor, MI, USA).

### Apoptosis assay and adhesion assays in human immortalized podocytes

Apoptosis assay was performed to examine the effects of serum starvation or high glucose. Human immortalized podocytes were cultured in RPMI 1640 medium (Wako) with 10% or 0.5% fetal bovine serum for 48 h for serum starvation. Human immortalized podocytes were cultured in RPMI 1640 medium (Wako) with 2000 or 5400 mg/dl for 48 h for high glucose. Then, cells were harvested for Annexin V-FITC/PI flow cytometry. Adhesion assay was performed using CytoSelect 48-well Cell Adhesion Assay (Collagen IV-coated, Colorimetric) kit (Cell Biolabs, San Diego, CA, USA). The OD 570 nm was measured in a plate reader.

### Statistical analyses

Data are represented as the mean ± standard deviation (SD). Statistical analyses of the data were performed using the StatView software program version 5.0 (SAS Institute, Cary, NC, USA). Comparisons among the three mouse groups were performed using a one-way analysis of variance, followed by Scheffe's multiple comparison test. Comparisons between two mouse groups were performed using Student's *t* test. *P* values of < 0.05 were considered significant.

## Supplementary Information

Supplementary Table S1.

Supplementary Table S2.

Supplementary Information.

## Data Availability

The datasets generated during and/or analysed during the current study are available from the corresponding author on reasonable request.

## References

[1] Gbadegesin R, Lavin P, Foreman J, Winn M (2011). Pathogenesis and therapy of focal segmental glomerulosclerosis: An update. Pediatr. Nephrol..

[2] D'Agati VD, Fogo AB, Bruijn JA, Jennette JC (2004). Pathologic classification of focal segmental glomerulosclerosis: A working proposal. Am. J. Kidney Dis..

[3] Tsuchimoto A (2020). Utility of Columbia classification in focal segmental glomerulosclerosis: Renal prognosis and treatment response among the pathological variants. Nephrol. Dial. Transplant..

[4] Rosenberg AZ, Kopp JB (2017). Focal segmental glomerulosclerosis. Clin. J. Am. Soc. Nephrol..

[5] McCarthy HJ (2013). Simultaneous sequencing of 24 genes associated with steroid-resistant nephrotic syndrome. Clin. J. Am. Soc. Nephrol..

[6] Sadowski, C. E. *et al.* A single-gene cause in 29.5% of cases of steroid-resistant nephrotic syndrome. *J. Am. Soc. Nephrol.* **26**, 1279–1289 (2015). 10.1681/ASN.2014050489 PMC4446877 25349199

[7] D'Agati VD, Kaskel FJ, Falk RJ (2011). Focal segmental glomerulosclerosis. N. Engl. J. Med..

[8] Knust E (1987). EGF homologous sequences encoded in the genome of *Drosophila**melanogaster*, and their relation to neurogenic genes. EMBO J..

[9] Tepass U (1996). Crumbs, a component of the apical membrane, is required for zonula adherens formation in primary epithelia of Drosophila. Dev. Biol..

[10] Bachmann A, Schneider M, Theilenberg E, Grawe F, Knust E (2001). Drosophila Stardust is a partner of Crumbs in the control of epithelial cell polarity. Nature.

[11] Médina E (2002). Crumbs interacts with moesin and beta(Heavy)-spectrin in the apical membrane skeleton of Drosophila. J. Cell Biol..

[12] Wei Z, Li Y, Ye F, Zhang M (2015). Structural basis for the phosphorylation-regulated interaction between the cytoplasmic tail of cell polarity protein crumbs and the actin-binding protein moesin. J. Biol. Chem..

[13] den Hollander AI (1999). Mutations in a human homologue of Drosophila crumbs cause retinitis pigmentosa (RP12). Nat. Genet..

[14] Li P, Mao X, Ren Y, Liu P (2015). Epithelial cell polarity determinant CRB3 in cancer development. Int. J. Biol. Sci..

[15] Ebarasi L (2009). A reverse genetic screen in the zebrafish identifies crb2b as a regulator of the glomerular filtration barrier. Dev. Biol..

[16] Xiao Z (2011). Deficiency in Crumbs homolog 2 (Crb2) affects gastrulation and results in embryonic lethality in mice. Dev. Dyn..

[17] Ebarasi L (2015). Defects of CRB2 cause steroid-resistant nephrotic syndrome. Am. J. Hum. Genet..

[18] Slavotinek A (2015). CRB2 mutations produce a phenotype resembling congenital nephrosis, Finnish type, with cerebral ventriculomegaly and raised alpha-fetoprotein. Am. J. Hum. Genet..

[19] Huber TB (2003). Molecular basis of the functional podocin-nephrin complex: Mutations in the NPHS2 gene disrupt nephrin targeting to lipid raft microdomains. Hum. Mol. Genet..

[20] Schwarz K (2001). Podocin, a raft-associated component of the glomerular slit diaphragm, interacts with CD2AP and nephrin. J. Clin. Investig..

[21] Lehtonen S, Zhao F, Lehtonen E (2002). CD2-associated protein directly interacts with the actin cytoskeleton. Am. J. Physiol. Renal Physiol..

[22] Roselli S (2004). Early glomerular filtration defect and severe renal disease in podocin-deficient mice. Mol. Cell Biol..

[23] Kerjaschki D, Sharkey DJ, Farquhar MG (1984). Identification and characterization of podocalyxin—The major sialoprotein of the renal glomerular epithelial cell. J. Cell Biol..

[24] Doyonnas R (2001). Anuria, omphalocele, and perinatal lethality in mice lacking the CD34-related protein podocalyxin. J. Exp. Med..

[25] Orlando RA (2001). The glomerular epithelial cell anti-adhesin podocalyxin associates with the actin cytoskeleton through interactions with ezrin. J. Am. Soc. Nephrol..

[26] Takeda T, McQuistan T, Orlando RA, Farquhar MG (2001). Loss of glomerular foot processes is associated with uncoupling of podocalyxin from the actin cytoskeleton. J. Clin. Investig..

[27] Vivante A (2011). Persistent asymptomatic isolated microscopic hematuria in Israeli adolescents and young adults and risk for end-stage renal disease. JAMA.

[28] Moreno JA (2012). Haematuria: The forgotten CKD factor?. Nephrol. Dial. Transplant..

[29] McCarthy ET, Sharma M, Savin VJ (2010). Circulating permeability factors in idiopathic nephrotic syndrome and focal segmental glomerulosclerosis. Clin. J. Am. Soc. Nephrol..

[30] Wei C (2011). Circulating urokinase receptor as a cause of focal segmental glomerulosclerosis. Nat. Med..

[31] Winnicki W (2019). Diagnostic and prognostic value of soluble urokinase-type plasminogen activator receptor (suPAR) in focal segmental glomerulosclerosis and impact of detection method. Sci. Rep..

[32] Suranyi MG, Guasch A, Hall BM, Myers BD (1993). Elevated levels of tumor necrosis factor-alpha in the nephrotic syndrome in humans. Am. J. Kidney Dis..

[33] Chen CL (2010). The apical-basal cell polarity determinant Crumbs regulates Hippo signaling in Drosophila. Proc. Natl. Acad. Sci. U.S.A..

[34] Michgehl U, Pavenstädt H, Vollenbröker B (2017). Cross talk between the Crumbs complex and Hippo signaling in renal epithelial cells. Pflugers Arch..

[35] Hamano, S., *et al.* Association of crumbs homolog-2 with mTORC1 in developing podocyte. *PLoS One* **13**, e0202400 (2018). 10.1371/journal.pone.0202400 PMC6101391 30125302

[36] Murata T (2016). COL4A6 is dispensable for autosomal recessive Alport syndrome. Sci. Rep..

[37] Katayama K (2008). Irradiation prolongs survival of Alport mice. J. Am. Soc. Nephrol..

[38] Takemoto M (2002). A new method for large scale isolation of kidney glomeruli from mice. Am. J. Pathol..

[39] Bechtel W (2013). Vps34 deficiency reveals the importance of endocytosis for podocyte homeostasis. J. Am. Soc. Nephrol..

[40] Kawachi H, Koike H, Kurihara H, Sakai T, Shimizu F (2003). Cloning of rat homologue of podocin: Expression in proteinuric states and in developing glomeruli. J. Am. Soc. Nephrol..

[41] Fukusumi Y (2015). SV2B is essential for the integrity of the glomerular filtration barrier. Lab. Investig..

[42] Putaala H, Soininen R, Kilpeläinen P, Wartiovaara J, Tryggvason K (2001). The murine nephrin gene is specifically expressed in kidney, brain and pancreas: Inactivation of the gene leads to massive proteinuria and neonatal death. Hum. Mol. Genet..

[43] Saleem MA (2002). A conditionally immortalized human podocyte cell line demonstrating nephrin and podocin expression. J. Am. Soc. Nephrol..

