# Supplementary material for: Podocyte-specific Crb2 knockout mice develop focal segmental glomerulosclerosis
Source: Sci Rep. 2021 Oct 15;11:20556. doi: 10.1038/s41598-021-00159-z (PMC8519956; doi:10.1038/s41598-021-00159-z)
Supplement: Supplementary file 3 — Supplementary Information. [file 41598_2021_159_MOESM3_ESM.pdf]

Supplementary Information

Podocyte-specific Crb2 knockout mice develop focal segmental glomerulosclerosis

Akiko Tanoue, MD[1,+], Kan Katayama, MD[1,2,+,*], Yugo Ito, MD[2,3], Kensuke Joh, MD[4], Masaaki Toda, PhD[5], Taro Yasuma, MD[5], Corina N D'Alessandro-Gabazza, MD[5], Hiroshi Kawachi, MD[6], Kunimasa Yan, MD[3], Masaaki Ito, MD[1], Esteban C Gabazza, MD[5], Karl Tryggvason, MD[2,7], Kaoru Dohi, MD[1]

Supplementary Methods

RNA-sequencing

Total RNA was extracted from the kidneys of Crb2$^{fl/fl}$ mice or Crb2$^{fl/fl}$pod-Cre$^{Tg/+}$ mice at six months of age (each $n = 5$) using a TRIzol Plus RNA purification kit (ThermoFisher Scientific). An Ion PI Chip Kit v3 (ThermoFisher Scientific) was used to perform sequencing with an Ion Proton Sequencer. The log 2 ratio was calculated from fragments per kilobase of exon per million reads mapped (FPKM) to compare the two groups. Comparisons between two mouse groups were performed using Student's *t*-test. *P* values of <0.05 were considered significant. *Q* value was calculated by Benjamini & Hochberg method to control the false discovery rate.

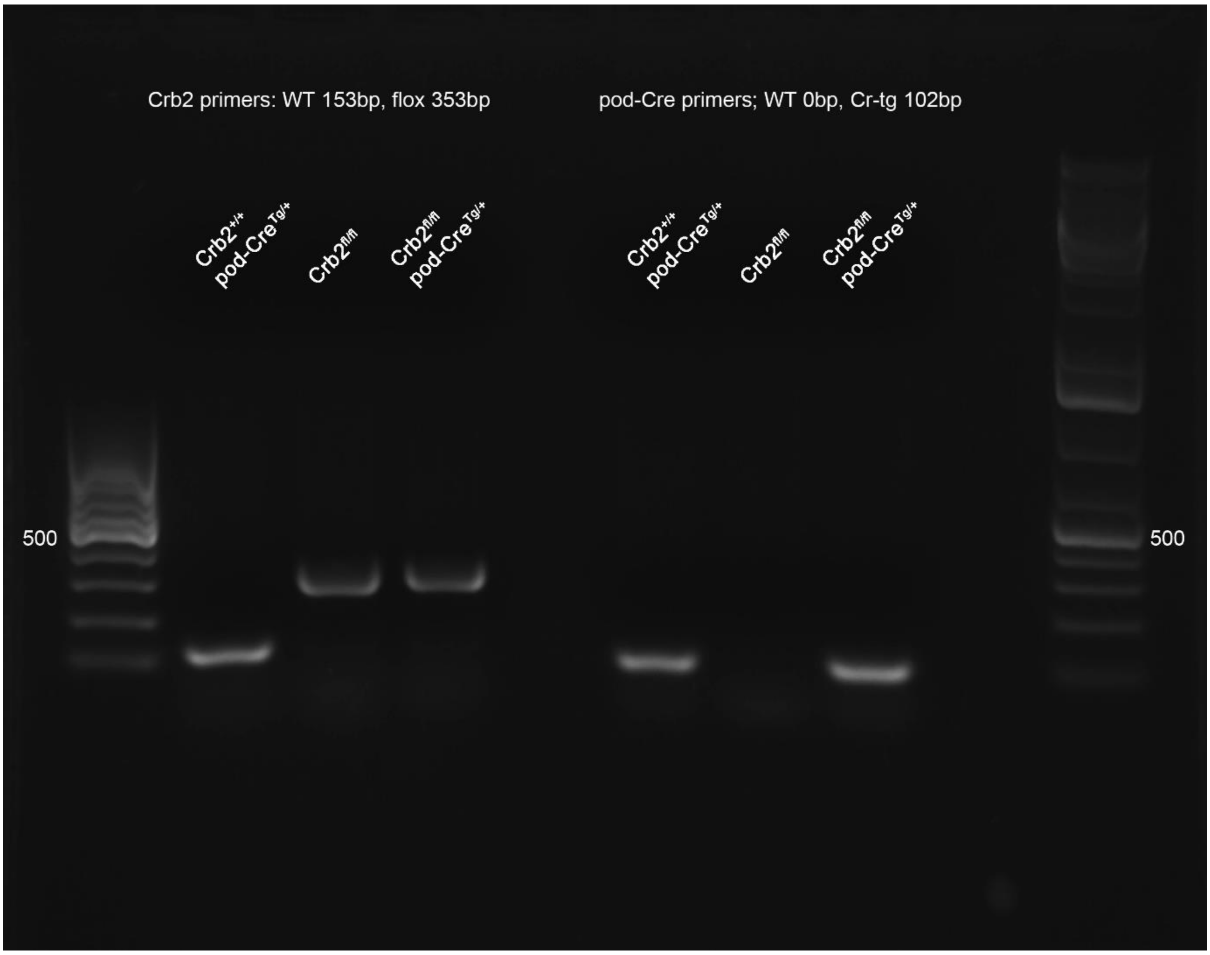

Supplementary Figure S1. Full length gels.

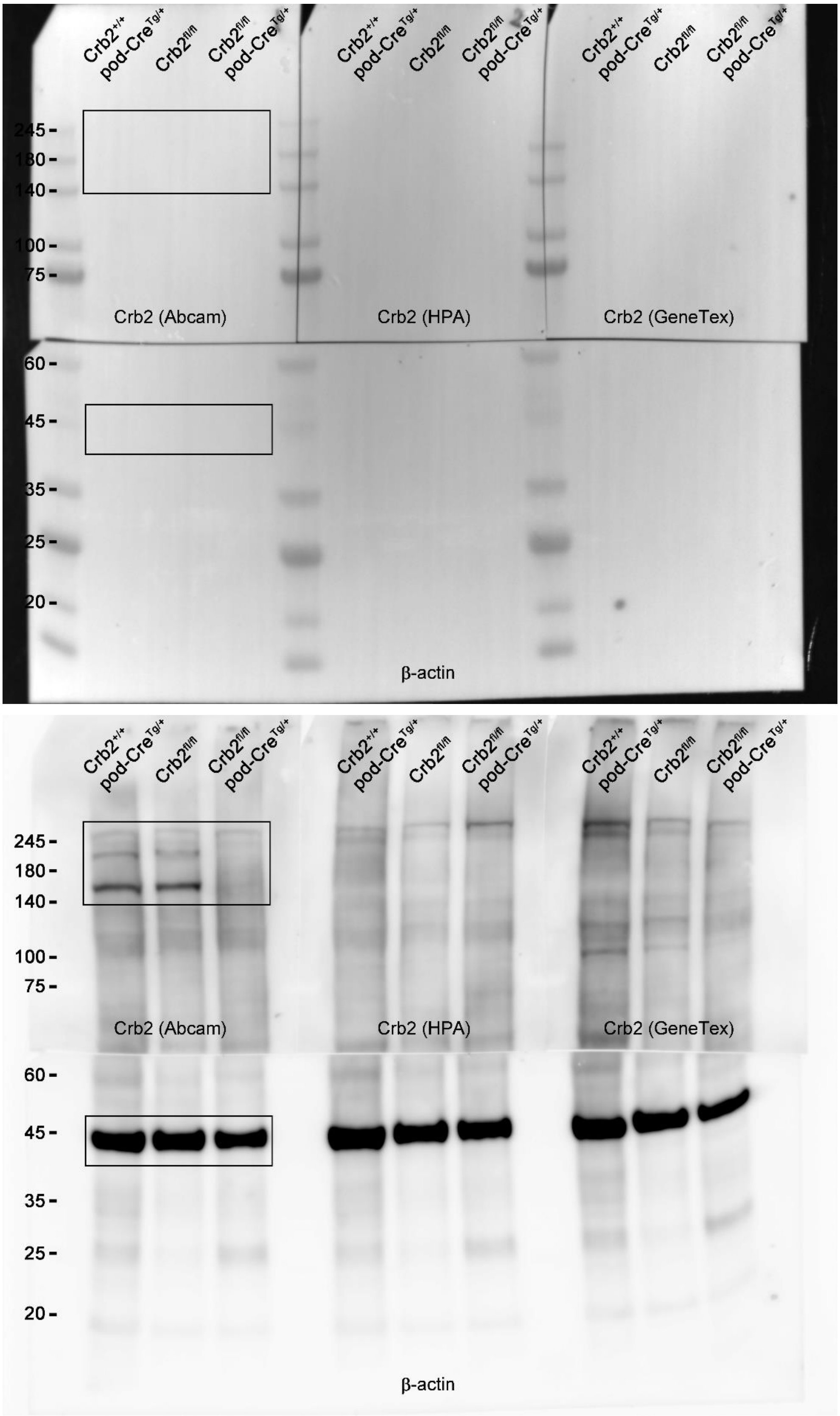

Supplementary Figure S2. Full length blots.

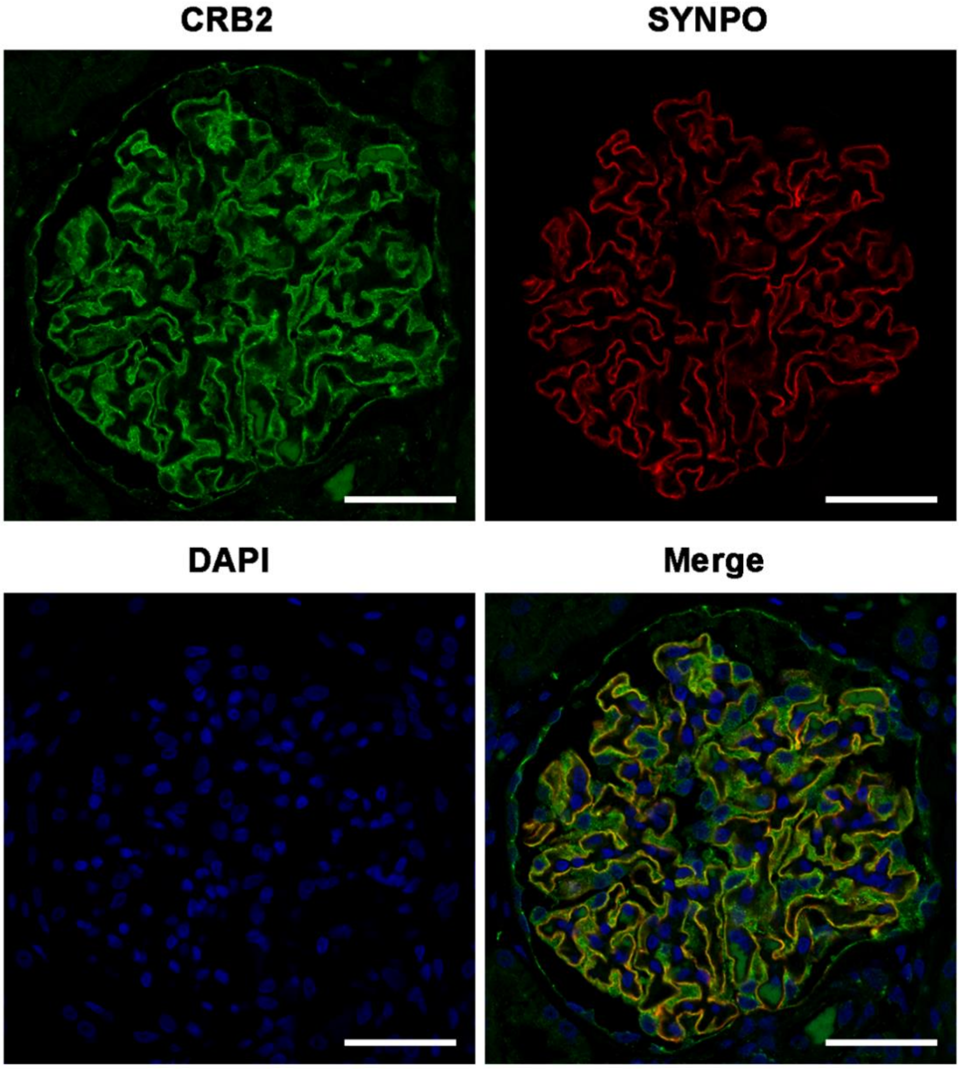

Supplementary Figure S3. Dual staining with CRB2 and SYNPO, a podocyte marker, in normal human paraffin kidney section showed colocalization of CRB2 and SYNPO. Scale bars 50 μm.

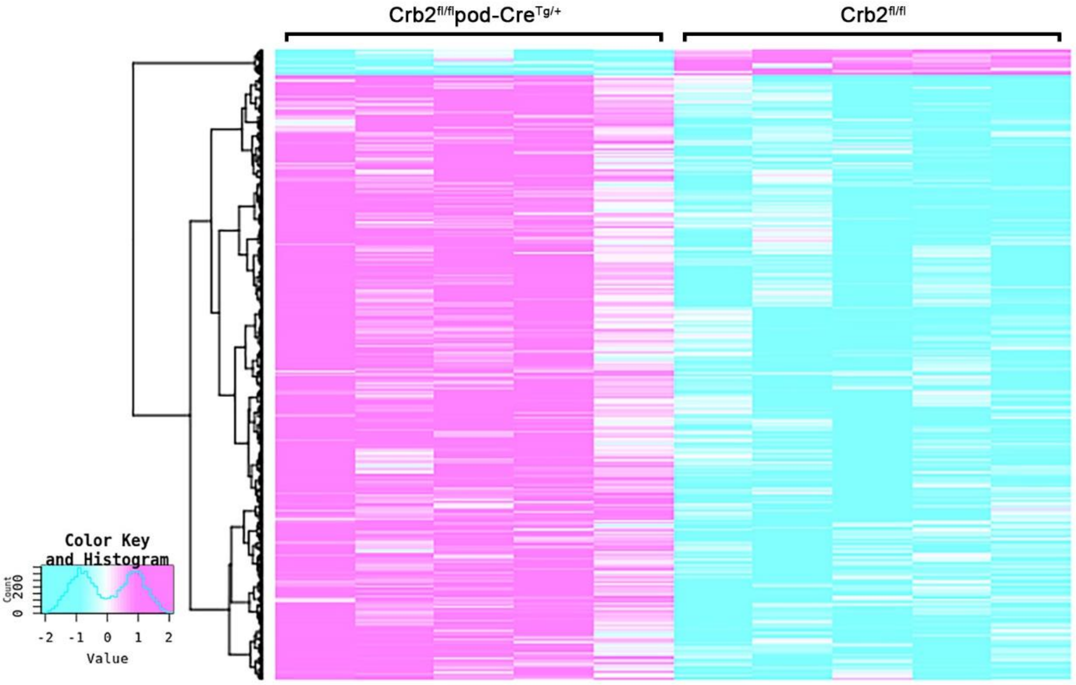

Supplementary Figure S4. The heat map from RNA-seq data after Benjamini & Hochberg method showed 666 differentially expressed genes between Crb2$^{fl/fl}$pod-Cre$^{Tg/+}$ mice and Crb2$^{fl/fl}$ mice.

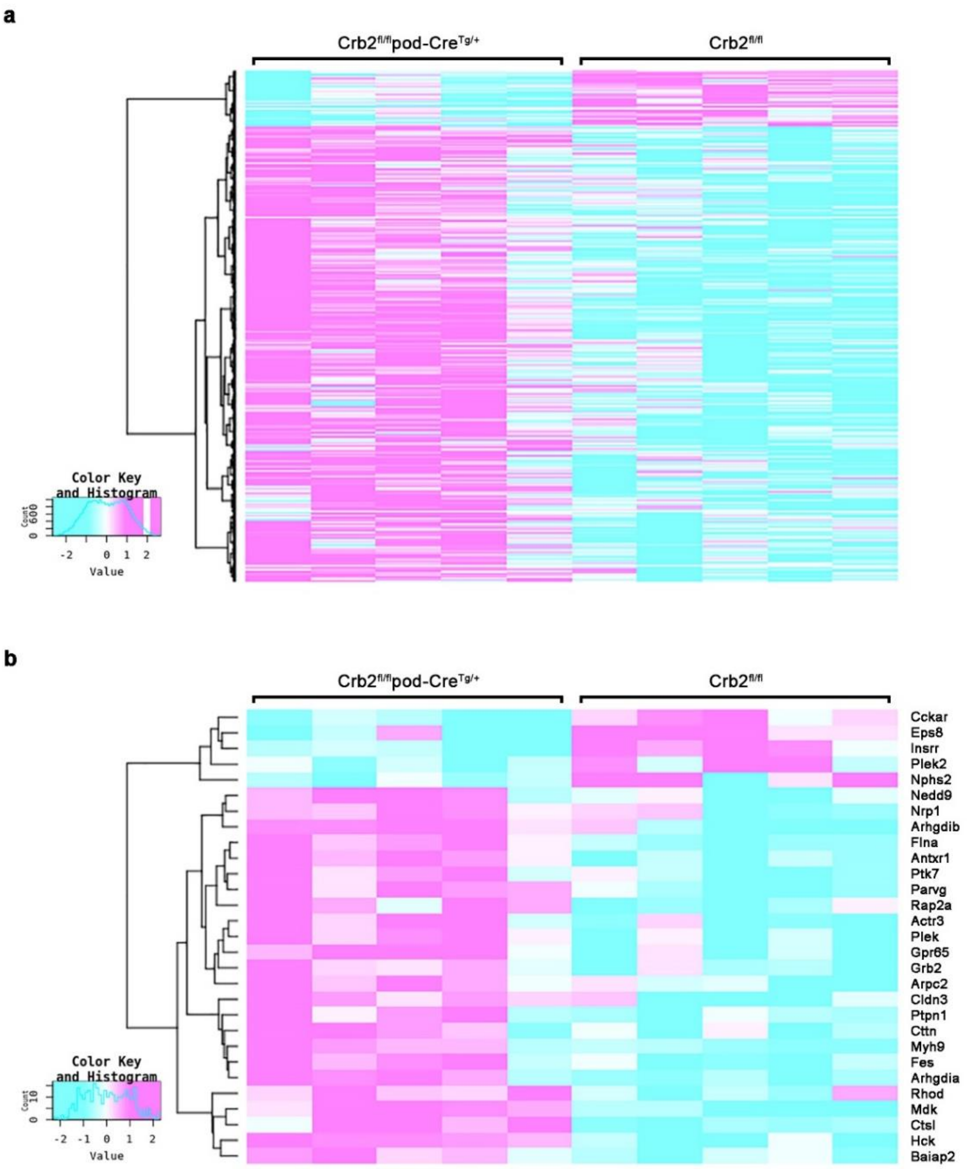

Supplementary Figure S5. RNA-seq analysis of the total kidney RNA extracts between Crb2$^{fl/fl}$pod-Cre$^{Tg/+}$ mice and Crb2$^{fl/fl}$ mice at six months of age. (a) The heat map from RNA-seq data before Benjamini & Hochberg method showed 3,174 differentially expressed genes between Crb2$^{fl/fl}$pod-Cre$^{Tg/+}$ mice and Crb2$^{fl/fl}$ mice. (b) The heat map from RNA-seq data showed 29 differentially expressed genes that were associated with actin cytoskeleton reorganization (GO: 0031532).

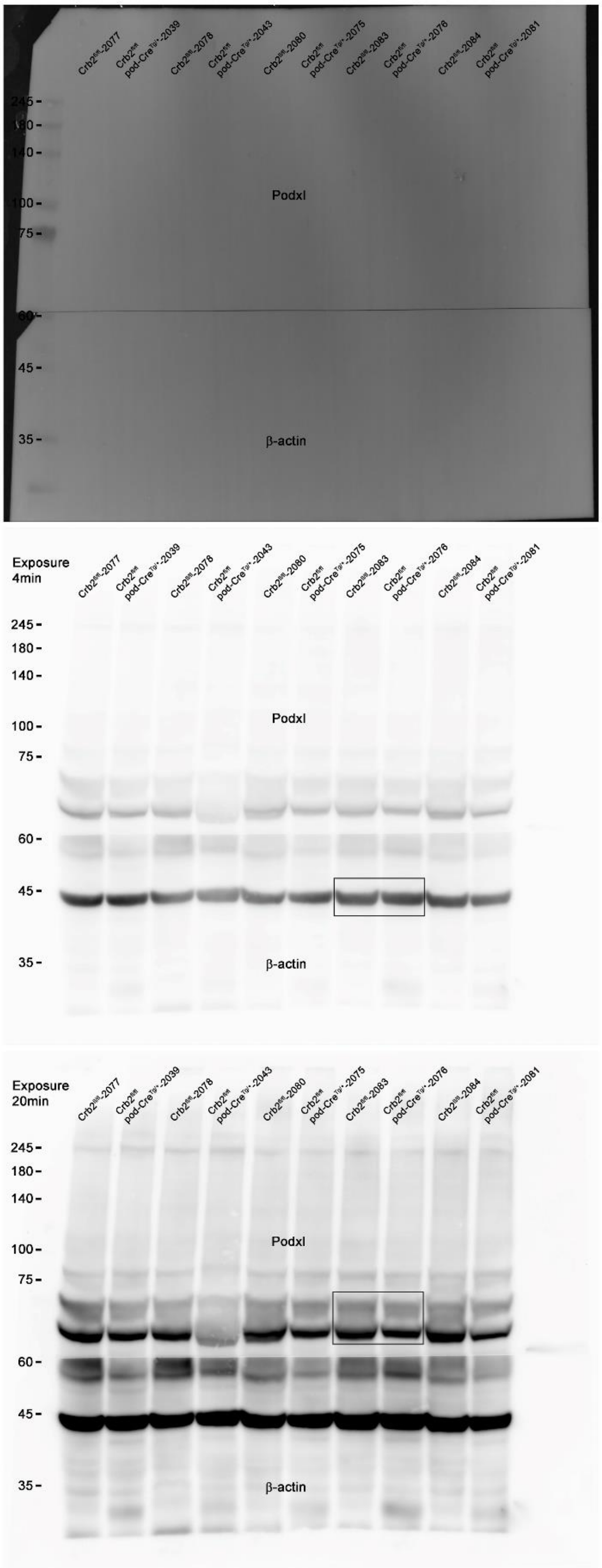

Supplementary Figure S6. Full length blots.

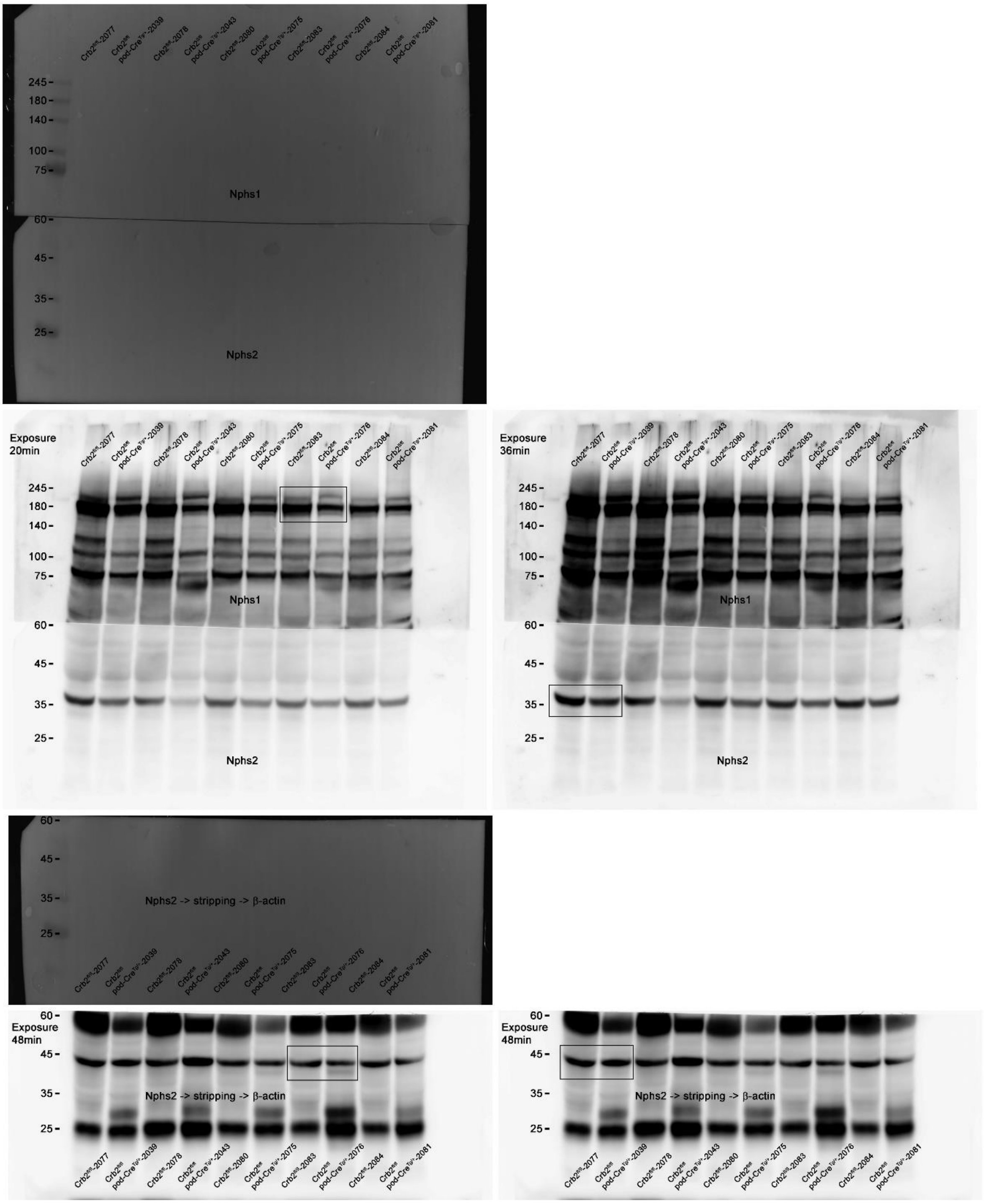

Supplementary Figure S7. Full length blots.

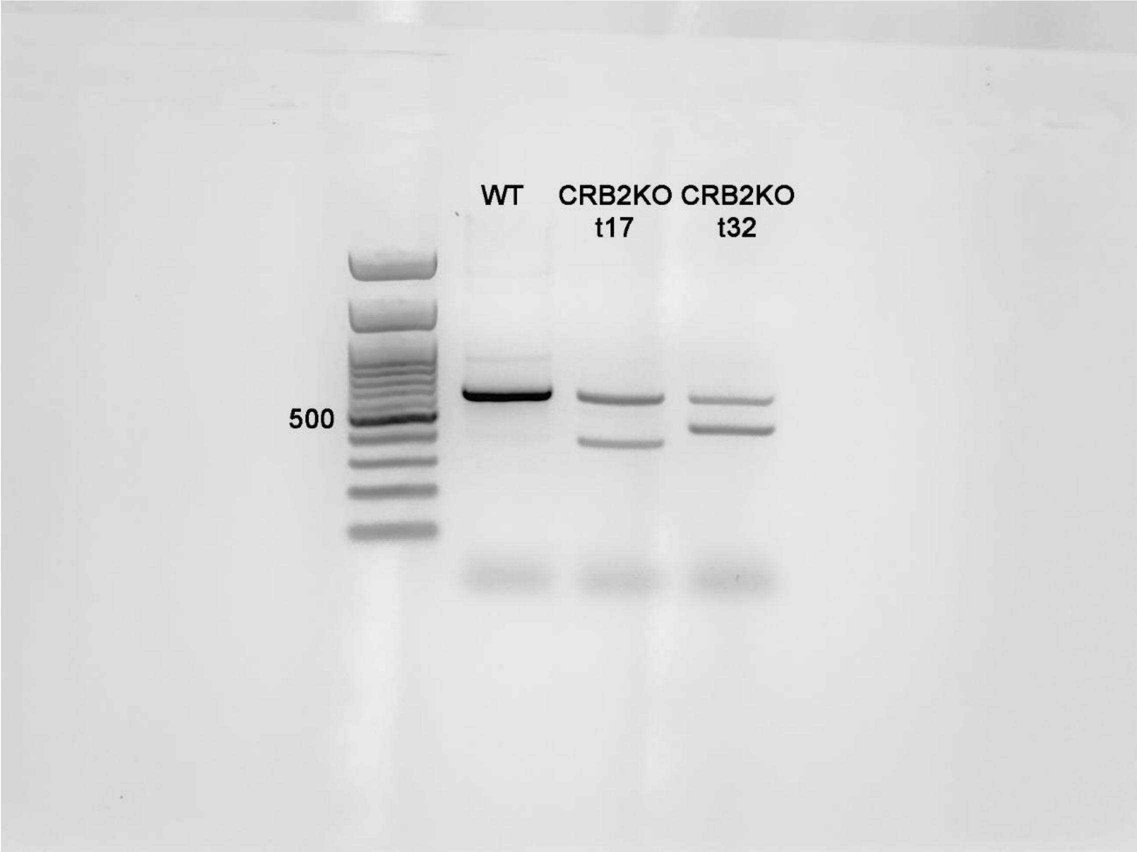

Supplementary Figure S8. Full length gels.

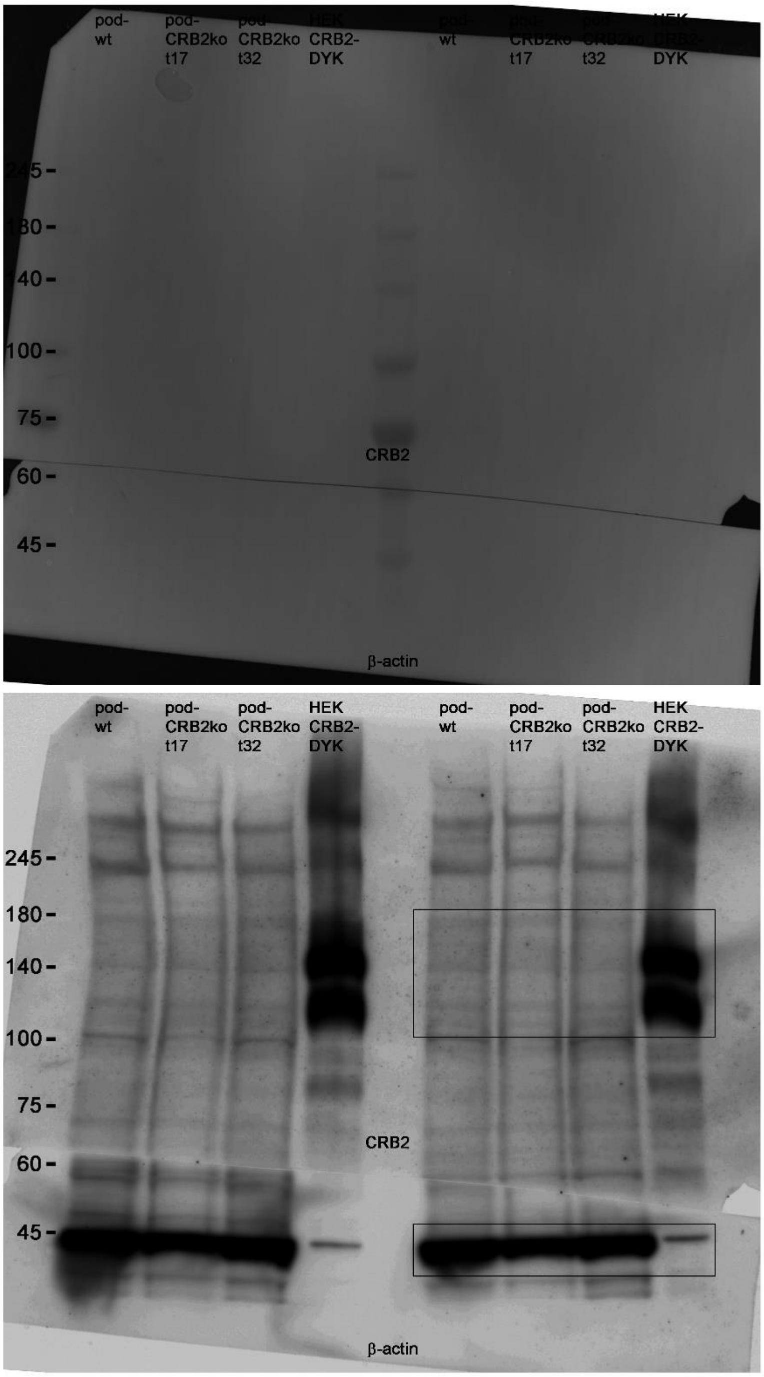

Supplementary Figure S9. Full length blots.

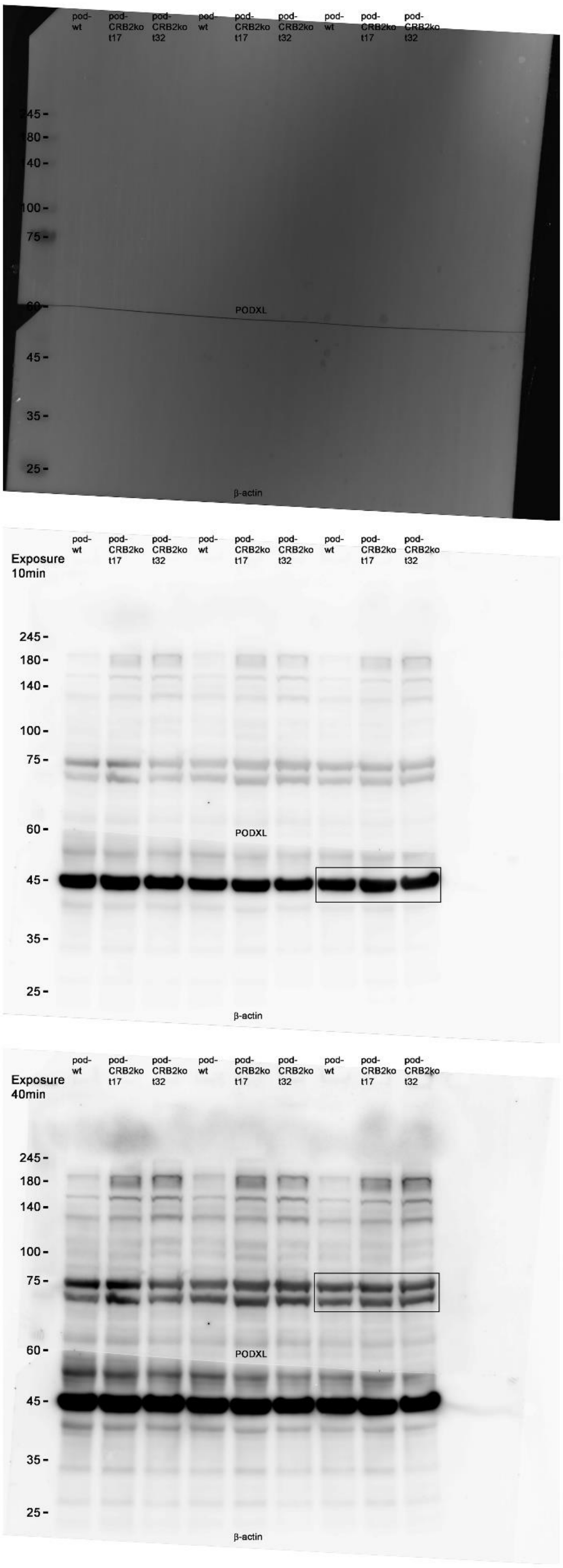

Supplementary Figure S10. Full length blots.

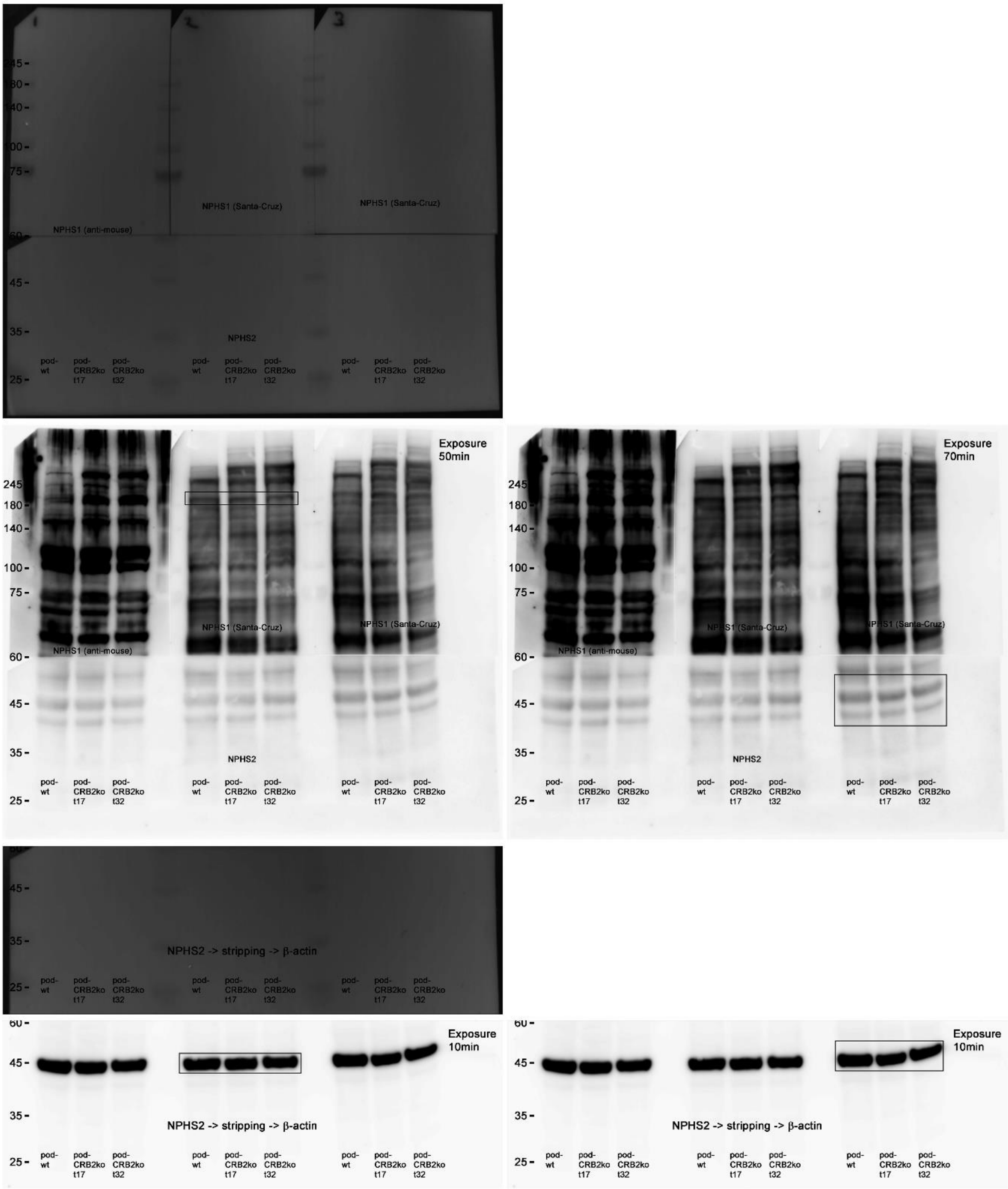

Supplementary Figure S11. Full length blots.

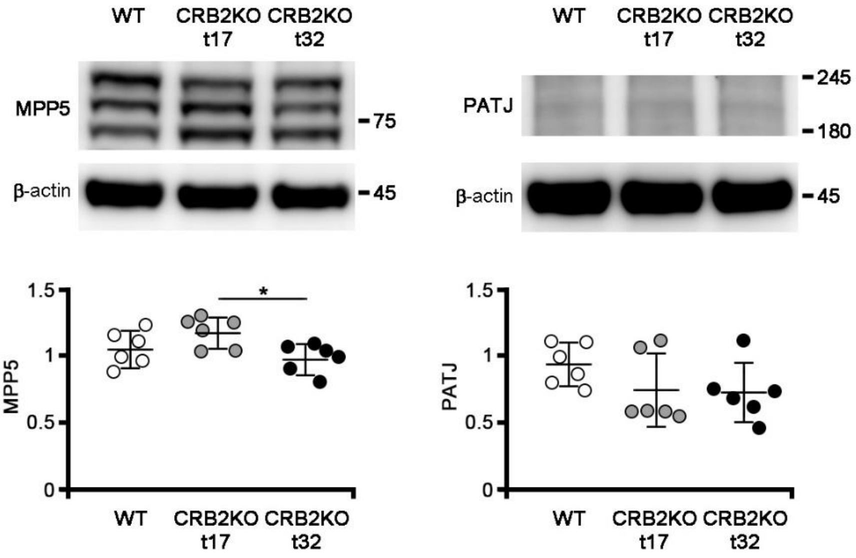

Supplementary Figure S12. Western blot analysis. The expressions of MPP5 (PALS1) and PATJ were comparable between wild-type and CRB2 knockout t17 or t32 clone. Data are presented as mean ± SD. Statistical analysis by one-way analysis of variance, followed by Scheffe's multiple comparison test; $n = 6$ in each group. $^*P < 0.05$. CRB2KO, CRB2 knockout; WT, wild-type.

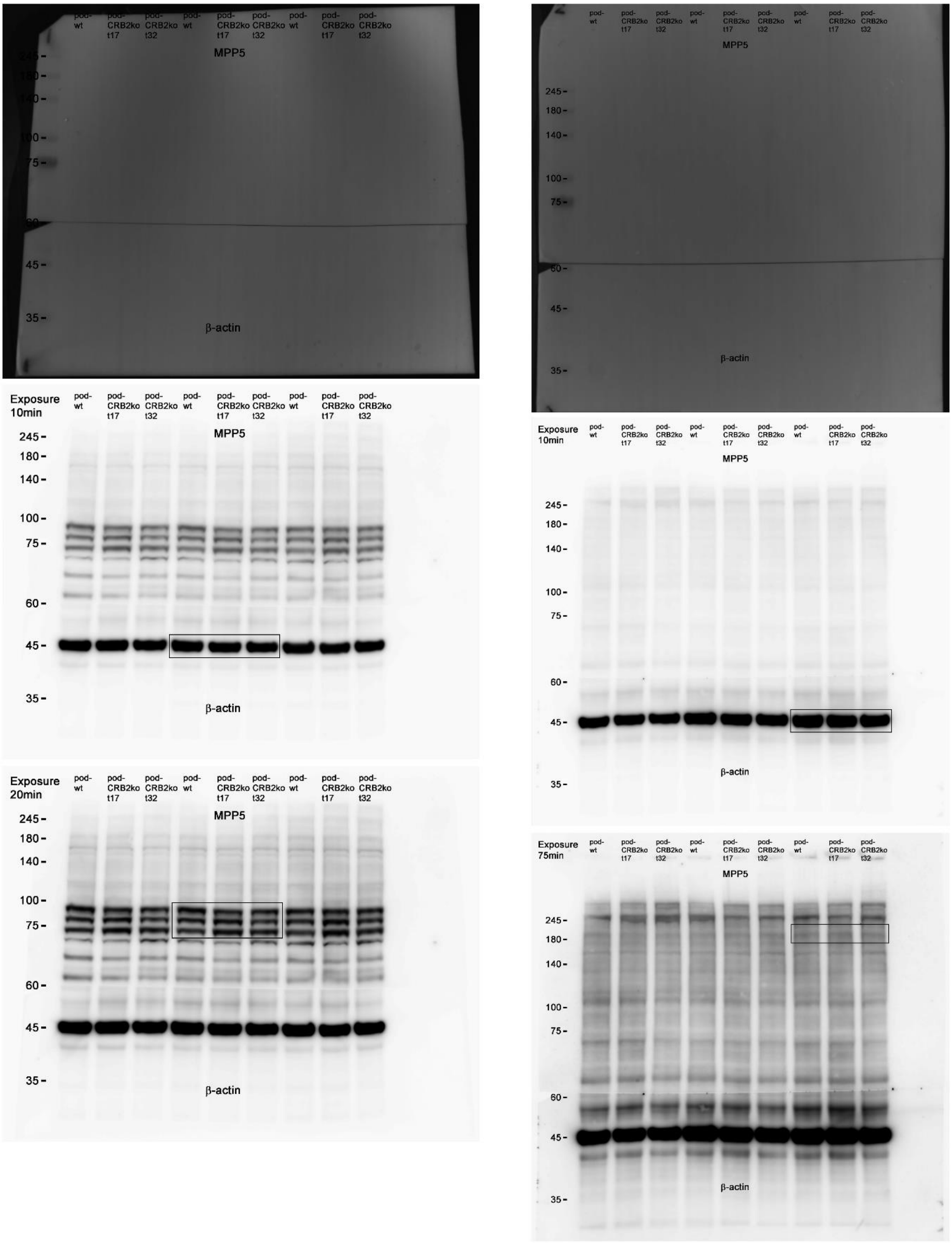

Supplementary Figure S13. Full length blots.

Supplementary Table S1. The FPKM of 23,913 genes (Crb2$^{fl/fl}$pod-Cre$^{Tg/+}$ mice vs. Crb2$^{fl/fl}$ mice).

Supplementary Table S2. The log 2 ratio was calculated from the 13,880 genes. There were 3174 and 666 significant genes before and after correction with Benjamini & Hochberg method. Among 666 significant genes, 477 genes were upregulated >2-fold, and 16 genes were downregulated >2-fold in Crb2fl/flpod-CreTg/+ mice compared to Crb2fl/fl mice. Among 3174 significant genes (P < 0.05), 1173 genes were upregulated >2-fold and 91 genes were downregulated >2-fold in Crb2$^{fl/fl}$pod-Cre$^{Tg/+}$ mice compared to Crb2$^{fl/fl}$ mice.

Supplementary Table S3. List of the top 50 upregulated genes (Crb2$^{fl/fl}$pod-Cre$^{Tg/+}$ mice vs. Crb2$^{fl/fl}$ mice).

Gene name	Fold change	P value
Gm8989	12.46829454	0.019298833
Nup62-il4i1	10.88126439	0.025637238
Rpl30	8.810643919	0.019165951
Hist2h2aa2	8.584899434	0.049745248
Gpnmb	7.024930476	2.10896E-06
Lyz1	5.562341462	4.62815E-06
Lcn2	5.414324374	0.000152463
C3	5.059644997	1.35012E-05
Igj	4.509676107	6.18876E-06
Mmp12	4.322855777	4.41511E-05
Lyz2	4.220226067	5.91434E-06
Tmem254c	4.120248557	0.023221483
C4b	3.895213675	0.000138062
Irf4	3.88440741	5.5963E-05
Gm13139	3.815498997	0.046302063
Socs3	3.769922819	4.22481E-05
Fgg	3.730008662	1.15862E-05
Ccl6	3.698088051	2.42672E-05
Ltbp2	3.636151228	0.001318297
Clec7a	3.512668839	1.27358E-05
Wfdc17	3.493613442	7.85691E-06
Cd52	3.390721291	9.06779E-06
Fgb	3.372863553	0.001027696
Serpina3f	3.338314847	3.19237E-05
Serpina3g	3.325789153	3.24867E-05
Fbn2	3.305146815	0.008370783
Ctss	3.272568716	1.46021E-05
Cd48	3.255894489	0.001291265
Itgam	3.245105157	0.003406965
Clec12a	3.216620358	1.5306E-05
Cd68	3.182653941	0.000644202
Lcp2	3.166086428	0.00021437

Fcer1g	3.152743139	0.000573947
Lilrb4	3.115325993	9.0503E-05
Slamf7	3.051969313	0.000193061
Cd22	3.042341904	0.000228756
Siglec1	3.015427564	0.000305182
Rac2	3.013688736	0.000978433
Tyrobp	3.008423352	0.000112662
Itgax	3.003315165	4.40734E-05
Sash3	2.99235193	8.99449E-05
Hpse	2.988746018	0.002831924
Cd53	2.973379266	1.28422E-05
Cd79b	2.97156853	0.010067433
Col8a1	2.935325535	0.000546585
Aif1	2.92249236	0.000753079
Ttr	2.901980772	0.000536631
Kcnn4	2.896472537	0.000147677
Pstpip1	2.882440254	0.002910066
Hck	2.874011216	0.000348022

Supplementary Table S4. List of the top 50 downregulated genes ($Crb2^{fl/fl}$pod-$Cre^{Tg/+}$ mice vs. $Crb2^{fl/fl}$ mice).

Gene name	Fold change	P value
Gm8989	-10.42890421	0.015210395
Igf2	-3.063794679	0.030509928
Hdc	-2.94931241	0.026868878
9030619P08Rik	-2.919414982	0.022654066
Slc22a7	-2.830913706	0.011120101
Cyp2d12	-2.64227592	0.002192773
Aqp4	-2.546164357	0.000161136
Slc10a2	-2.497432276	6.10137E-08
Dnase1	-2.228894892	0.003900844
Bhmt	-2.177836854	0.017069458
Myh1	-2.103439592	0.025307765
Slc22a26	-1.912100221	0.0002472
Gnmt	-1.891678683	0.00668659
Akr1c14	-1.877825864	0.03115562
Slc22a27	-1.825775221	0.007884798
Mep1b	-1.800422647	0.00209396
Slitrk6	-1.771260456	0.001658349
Hao2	-1.752540101	0.039642322
4921530L18Rik	-1.695141139	0.007050611
Cpb2	-1.676523042	0.008871115
Hsd17b2	-1.669216308	0.00631797
Sec14l3	-1.642193034	0.025894067
1110032F04Rik	-1.626213396	0.006080414
4932435O22Rik	-1.612135076	0.022717182
Gm853	-1.58312922	0.013718506
6330410L21Rik	-1.525642044	0.025284721
C8a	-1.500393718	0.004442203
Egf	-1.499992406	0.046802799
Ell3	-1.490529679	0.001330616
Kap	-1.488228403	0.024840624
Gk2	-1.472050102	0.021314165
Nphs2	-1.464949514	0.047828899

Tfrc	-1.458133359	0.001945646
Ces2b	-1.435675268	0.025473172
Cyp2c44	-1.412157704	0.016932267
Slc16a4	-1.40961605	0.014485248
C730027H18Rik	-1.37284126	0.028764597
Pm20d1	-1.353774637	0.000239712
Fgfbp1	-1.337002325	0.00029839
6430571L13Rik	-1.335425963	0.003887843
Acy1	-1.334106933	0.011753335
D630029K05Rik	-1.283578326	0.008164409
Cndp1	-1.271703347	0.009293082
Fam107a	-1.245175642	0.006932568
Gpm6a	-1.232569392	0.003502323
Acsm1	-1.228582575	0.02615072
2310042D19Rik	-1.222477157	0.006290261
Akr1d1	-1.218862439	0.007637618
Dmgdh	-1.215981586	0.009296368
Abca13	-1.212699214	0.029659218

Supplementary Table S5. KEGG pathways in David functional analysis from 1173 more than two-fold upregulated genes (Crb2$^{fl/fl}$pod-Cre$^{Tg/+}$ mice vs. Crb2$^{fl/fl}$ mice).

Category	Term	Count	P-Value	Benjamini
KEGG_PATHWAY	Systemic lupus erythematosus	47	9.50E-20	2.30E-17
KEGG_PATHWAY	Staphylococcus aureus infection	27	4.70E-18	5.70E-16
KEGG_PATHWAY	Osteoclast differentiation	37	2.90E-14	2.40E-12
KEGG_PATHWAY	Phagosome	39	2.80E-11	1.70E-09
KEGG_PATHWAY	Leishmaniasis	23	6.00E-11	3.00E-09
KEGG_PATHWAY	Cell adhesion molecules (CAMs)	36	4.00E-10	1.60E-08
KEGG_PATHWAY	Tuberculosis	36	4.20E-09	1.50E-07
KEGG_PATHWAY	ECM-receptor interaction	24	9.70E-09	3.00E-07
KEGG_PATHWAY	Fc gamma R-mediated phagocytosis	23	2.00E-08	5.30E-07
KEGG_PATHWAY	HTLV-I infection	45	5.30E-08	1.30E-06
KEGG_PATHWAY	Viral carcinogenesis	40	6.50E-08	1.40E-06
KEGG_PATHWAY	Complement and coagulation cascades	21	8.00E-08	1.60E-06
KEGG_PATHWAY	TNF signaling pathway	25	1.70E-07	3.00E-06
KEGG_PATHWAY	Amoebiasis	26	1.70E-07	3.00E-06
KEGG_PATHWAY	Focal adhesion	36	3.00E-07	4.90E-06
KEGG_PATHWAY	B cell receptor signaling pathway	19	5.20E-07	8.00E-06
KEGG_PATHWAY	Leukocyte transendothelial migration	25	7.90E-07	1.10E-05
KEGG_PATHWAY	Epstein-Barr virus infection	27	9.60E-07	1.30E-05
KEGG_PATHWAY	Natural killer cell mediated cytotoxicity	23	1.30E-06	1.70E-05
KEGG_PATHWAY	Transcriptional misregulation in cancer	30	1.40E-06	1.70E-05
KEGG_PATHWAY	Platelet activation	25	5.50E-06	6.40E-05
KEGG_PATHWAY	Chemokine signaling pathway	32	6.20E-06	6.90E-05
KEGG_PATHWAY	Toll-like receptor signaling pathway	21	1.00E-05	1.10E-04
KEGG_PATHWAY	Alcoholism	32	1.20E-05	1.20E-04
KEGG_PATHWAY	Hepatitis B	26	1.20E-05	1.20E-04
KEGG_PATHWAY	Intestinal immune network for IgA production	13	1.30E-05	1.20E-04
KEGG_PATHWAY	Antigen processing and presentation	18	2.50E-05	2.20E-04
KEGG_PATHWAY	Influenza A	28	2.50E-05	2.20E-04
KEGG_PATHWAY	Inflammatory bowel disease (IBD)	15	2.60E-05	2.20E-04
KEGG_PATHWAY	Herpes simplex infection	31	5.40E-05	4.40E-04
KEGG_PATHWAY	Viral myocarditis	17	5.80E-05	4.60E-04
KEGG_PATHWAY	Pertussis	16	9.70E-05	7.40E-04
KEGG_PATHWAY	Cytokine-cytokine receptor interaction	33	1.70E-04	1.20E-03

KEGG_PATHWAY	Toxoplasmosis	19	2.00E-04	1.40E-03
KEGG_PATHWAY	NF-kappa B signaling pathway	18	2.20E-04	1.60E-03
KEGG_PATHWAY	Measles	22	2.70E-04	1.90E-03
KEGG_PATHWAY	Primary immunodeficiency	10	3.00E-04	2.00E-03
KEGG_PATHWAY	Rheumatoid arthritis	16	3.20E-04	2.10E-03
KEGG_PATHWAY	T cell receptor signaling pathway	18	3.70E-04	2.30E-03
KEGG_PATHWAY	DNA replication	10	3.90E-04	2.40E-03
KEGG_PATHWAY	PI3K-Akt signaling pathway	41	6.50E-04	3.90E-03
KEGG_PATHWAY	Asthma	8	7.60E-04	4.40E-03
KEGG_PATHWAY	Regulation of actin cytoskeleton	28	1.00E-03	5.90E-03
KEGG_PATHWAY	Allograft rejection	12	1.10E-03	6.00E-03
KEGG_PATHWAY	MAPK signaling pathway	31	1.40E-03	7.90E-03
KEGG_PATHWAY	Hematopoietic cell lineage	15	1.50E-03	7.90E-03
KEGG_PATHWAY	Jak-STAT signaling pathway	21	1.60E-03	8.50E-03
KEGG_PATHWAY	Graft-versus-host disease	11	2.10E-03	1.10E-02
KEGG_PATHWAY	Malaria	10	4.20E-03	2.10E-02
KEGG_PATHWAY	Legionellosis	11	4.30E-03	2.10E-02
KEGG_PATHWAY	Proteoglycans in cancer	25	4.80E-03	2.30E-02
KEGG_PATHWAY	Fc epsilon RI signaling pathway	12	5.30E-03	2.50E-02
KEGG_PATHWAY	Protein digestion and absorption	14	5.70E-03	2.60E-02
KEGG_PATHWAY	Autoimmune thyroid disease	12	7.40E-03	3.40E-02
KEGG_PATHWAY	Type I diabetes mellitus	11	7.90E-03	3.50E-02
KEGG_PATHWAY	HIF-1 signaling pathway	15	8.10E-03	3.60E-02
KEGG_PATHWAY	Cytosolic DNA-sensing pathway	11	9.90E-03	4.20E-02
KEGG_PATHWAY	Colorectal cancer	11	9.90E-03	4.20E-02
KEGG_PATHWAY	Small cell lung cancer	13	1.00E-02	4.20E-02
KEGG_PATHWAY	Acute myeloid leukemia	10	1.20E-02	4.80E-02
KEGG_PATHWAY	Salmonella infection	12	1.50E-02	5.90E-02
KEGG_PATHWAY	Pathways in cancer	39	1.60E-02	6.10E-02
KEGG_PATHWAY	Rap1 signaling pathway	24	1.70E-02	6.70E-02
KEGG_PATHWAY	Apoptosis	10	1.80E-02	6.90E-02
KEGG_PATHWAY	MicroRNAs in cancer	29	1.90E-02	7.10E-02
KEGG_PATHWAY	Cell cycle	16	1.90E-02	7.10E-02
KEGG_PATHWAY	Hepatitis C	17	2.00E-02	7.30E-02
KEGG_PATHWAY	Chagas disease (American trypanosomiasis)	14	2.00E-02	7.30E-02
KEGG_PATHWAY	Gap junction	12	2.90E-02	1.00E-01
KEGG_PATHWAY	Glioma	10	2.90E-02	1.00E-01
KEGG_PATHWAY	NOD-like receptor signaling pathway	9	3.60E-02	1.20E-01

KEGG_PATHWAY	Progesterone-mediated oocyte maturation	11	6.70E-02	2.30E-01
KEGG_PATHWAY	Prion diseases	6	6.80E-02	2.30E-01
KEGG_PATHWAY	Pancreatic cancer	9	6.90E-02	2.30E-01
KEGG_PATHWAY	Glycosphingolipid biosynthesis - ganglio series	4	7.50E-02	2.50E-01
KEGG_PATHWAY	Choline metabolism in cancer	12	7.70E-02	2.50E-01
KEGG_PATHWAY	Non-small cell lung cancer	8	8.00E-02	2.60E-01
KEGG_PATHWAY	Axon guidance	14	9.30E-02	2.90E-01
KEGG_PATHWAY	mTOR signaling pathway	8	1.00E-01	3.10E-01

Supplementary Table S6. Urine albumin-to-creatinine ratio at one month of age.

Mouse	Urine albumin-to-creatinine ratio (μg/mg·Cr)
$Crb2^{fl/fl}$	29
$Crb2^{fl/fl}$	59
$Crb2^{fl/+}$pod-$Cre^{Tg/+}$	61
$Crb2^{fl/fl}$pod-$Cre^{Tg/+}$	84291
